# New Insights into the *In Situ* Fenton-like Process by Copper(II)-hydroxylamine Coupling: Reactive Species, Applications, and Limitations

**DOI:** 10.1021/acsestwater.5c00913

**Published:** 2026-01-27

**Authors:** Simone Pellegrino, Pablo Martínez-Marco, Javier Moreno-Andrés, Esther Bautista-Chamizo, Ana María Amat, Claudio Minero, Enzo Laurenti, Iván Sciscenko, Marco Minella

**Affiliations:** 1 Department of Chemistry, 9314 University of Turin, Via Pietro Giuria 7, Turin 10125, Italy; 2 Textile and Paper Engineering Department, 16774 Universitat Politècnica de València, Plaza Ferrándiz y Carbonell S/N, Alcoy 03801, Spain; 3 Department of Environmental Technologies, Faculty of Marine and Environmental Sciences, INMAR-Marine Research Institute, University of Cádiz, Campus Universitario Puerto Real, Cádiz, Puerto Real 11510, Spain

**Keywords:** advanced oxidation processes, contaminants of emerging concern, trivalent copper, reactive nitrogen species, hard water, wastewater treatment

## Abstract

This work studied the effect of pH, [O_2_], H_2_O_2_ external addition, chelating agents, common anions, transition metals, and produced toxicity, in the process Cu­(II)/NH_2_OH (employing benzoate as a probe). It was observed that (i) acidification by NH_2_OH·HCl addition hindered the process significantly as the Cu­(NH_2_OH)^2+^ is principally formed at pH = 6–8, (ii) high HCO_3_^–^ concentrations (≥1 mM) accelerated the NH_2_OH consumption (>95% in 5 min) and pollutant oxidation (*k*_obs_ ≈ 2 × 10^–2^ min^–1^ with initial benzoate concentration 50 μM) due to the buffer effect and the formation of a Fenton-like active species, CuCO_3(aq)_, (iii) the Mo­(VI)-peroxo species formed by Mo­(VI) reaction with the generated H_2_O_2_ consumed the NH_2_OH, being a strong interference, and (iv) NH_2_OH, although mainly decomposed into gaseous products, remained in trace concentrations, exhibiting toxicity. Results with selective scavenger addition, electron paramagnetic resonance, NO_(g)_ kinetics, and high resolution mass spectrometry suggested that NH_2_OH not only acts as a Cu­(II) reducing agent (responsible for starting the Fenton reaction due to the *in situ* formation of H_2_O_2_ by Cu­(I)/O_2_ reaction), but it also changes the Fenton mechanism of Cu­(I, II)/H_2_O_2_, suggesting that the formed reactive nitrogen species are responsible for pollutant abatement rather than HO^•^ or Cu­(III).

## Graphical abstract

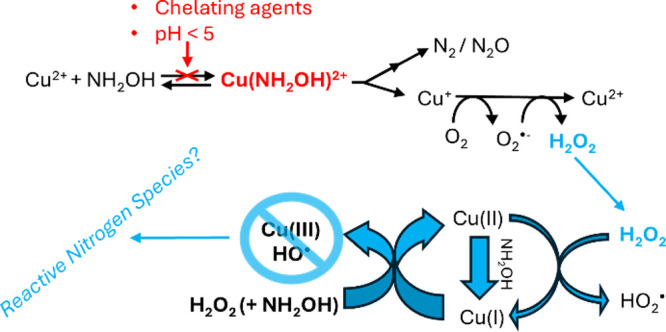

## 1 Introduction

Advanced oxidation processes (AOPs) have been largely proposed as an effective technology for water treatment. The generated reactive species can deal with emerging contaminants (e.g., pharmaceuticals), microorganisms, and antibiotic-resistant bacteria and genes.^1^ Within the aforementioned reactive species, the hydroxyl radical (HO^•^) is one of the most oxidative, not only from the thermodynamic point of view (*E*°(HO^•^/H_2_O) = 2.8 V vs NHE) but also from the kinetic one, being its reactivity usually limited by the reagent diffusion only (its bimolecular kinetic rate constant with most compounds is usually higher than 10^9^ M^–1^ s^–1^).

HO^•^ is produced in several AOPs, such as the well-known Fenton reaction (Fe­(II)/H_2_O_2_).^2,3^ Noteworthy, due to the limitations of the Fenton process (lack of pollutant abatement efficiency at pH > 4), replacing iron ions with copper ones to activate H_2_O_2_, or other peroxides, has been garnering interest in the past decade because of the broader pH applicability with the latter.^4–7^ The involved mechanism is stated to be similar to that with iron, in which the reaction between hydrogen peroxide and the metal’s lower oxidation state (R1, Cu­(I)/H_2_O_2_) is considerably faster than the one with the higher oxidation state (R2, Cu­(II)/H_2_O_2_).^8,9^ Therefore, enhancing the Cu­(II) reduction eventually implies faster pollutant removals by increasing the catalytic nature of the overall process.

$${\mathrm{Cu}}^{+}+H_{2}O_{2}\to {\mathrm{Cu}}^{2+}+{\mathrm{HO}}^{•}+{\mathrm{OH}}^{-}\;(\mathrm{or}\;{\mathrm{Cu}}^{3+}+2{\mathrm{OH}}^{-});k=4\times 10^{5}\;M^{-1}\;s^{-1}$$ R1

$${\mathrm{Cu}}^{2+}+H_{2}O_{2}\to {\mathrm{Cu}}^{+}+{{\mathrm{HO}}_{2}}^{•}+H^{+};\;k<1\;M^{-1}\;s^{-1}$$ R2

Hydroxylamine (NH_2_OH) is a common reducing agent employed for organic synthesis and an important intermediate during the biogeochemical nitrogen cycle.^10^ Recently, it has been gaining momentum as a cocatalyst of Fenton reactions driven by iron and/or copper, as it accelerates the above-mentioned catalytic loop.^11–15^ Lee et al.^16^ were the first to propose the Cu­(II)/NH_2_OH couple as a promising AOP for the removal of recalcitrant contaminants in water, with the inherent advantage of H_2_O_2_ *in situ* generation through the reaction between formed Cu­(I) and dissolved oxygen (see reactions, [Disp-formula eqR3]–[Disp-formula eqR5]). This is a relevant attribute since H_2_O_2_ is purchased chiefly as a concentrated liquid solution, which is inconvenient toward its transporting, handling, and storing, whereas NH_2_OH is sold as “pure” solid hydroxylammonium salt, making it easier to manage. Noteworthy, since the publication of the former work, few researchers have explored this strategy (with no peroxide addition).^13^ Furthermore, there is still no consensus on the involved mechanism governing Cu­(II)-reducing agent (or, most precisely, copper-based AOPs in general): several authors have proposed that, instead of HO^•^ (or SO_4_^•–^ if combined with persulfate or peroxymonosulfate), Cu­(III) is the main responsible for pollutant oxidation on Cu-based AOPs.^7,17,18^ The increasing interest in investigating Cu­(III) oxidation ability is reflected in recent studies synthesizing stable Cu­(III)-complexes to evaluate their reactivity against emerging contaminants.^19,20^ Other authors have also reported a significant contribution of singlet oxygen (^1^O_2_) toward pollutant abatement in copper-based AOPs.^21–23^ Finally, only a few have considered the potential role of reactive nitrogen species (RNS) formed if NH_2_OH is used as a reducing agent or as a reagent to activate peroxides, such as aminoxyl radical (NH_2_O^•^).^24,25^

$${\mathrm{Cu}}^{2+}+{\mathrm{NH}}_{2}\mathrm{OH}\to {\mathrm{Cu}}^{+}+1/2N_{2}+H_{2}O+H^{+}$$ R3

$${\mathrm{Cu}}^{+}+O_{2}\to {O_{2}}^{•-}+{\mathrm{Cu}}^{2+};\;k=3\times 10^{4}\;M^{-1}\;s^{-1}$$ R4

$${\mathrm{Cu}}^{+}+{O_{2}}^{•-}+2H^{+}\to H_{2}O_{2}+{\mathrm{Cu}}^{2+};\;k=2\times 10^{9}\;M^{-1}\;s^{-1}$$ R5

In addition to the mechanistic point of view involving Cu­(II)-reducing agent AOPs, with or without external peroxide addition, studies involving the effect of water constituents (dissolved organic matter and anions), metal chelating agents, or niche applications where to employ this treatment are still scarce.^26,27^ Furthermore, in these works, the effect of other transition metals (e.g., Fe­(III), Mn­(II), or Mo­(VI)), which can be present in the wastewater or intentionally added,^28–32^ is usually not considered. Based on this background, we have studied the scarcely explored Cu­(II)/NH_2_OH process with the intention of employing NH_2_OH as the only reagent required to treat wastewater, using benzoic acid (BA) as a probe, a compound with a high occurrence in wastewater effluents due to its natural and anthropogenic sources and typically used to evaluate AOP performances.^16,33–35^ The possible involved reactive species (e.g., HO^•^, Cu­(III), ^1^O_2_, NH_2_O^•^), effects of other water constituents and their interaction with copper, the generated oxidation byproducts, and the toxicity of the final effluent were investigated. The treatment was finally studied in simulated wastewater containing Cu­(II) in trace amounts. As far as we know, such an in-depth investigation of this process has never been reported before, which constitutes the main novelty of the present work.

## 2 Materials and Methods

### 2.1 Reagents and Analytical Measurements

CuSO_4_·5H_2_O, Fe­(ClO_4_)_3_·*x*H_2_O, FeCl_3_·6H_2_O, FeSO_4_·6H_2_O, MnSO_4_·H_2_O, Na_2_MoO_4_·2H_2_O, NH_2_OH·HCl, benzoic acid, phenol, *p*-benzoquinone, furfuryl alcohol, ascorbic acid, neocuproine, 1,10-phenanthroline, 4-aminopyridine, rose Bengal, horseradish peroxidase, 5,5-dimethyl-1-pyrroline *N*-oxide, 2,2,6,6-tetramethylpiperidine, ethylenediaminetetraacetic acid, and diethyldithiocarbamate were purchased from Sigma-Aldrich. Humic acid sodium salt, sodium acetate, KCl, NaIO_4_, Na_2_B_4_O_7_·10H_2_O, CaSO_4_·2H_2_O, MgSO_4_·7H_2_O, ZnSO_4_·H_2_O, NaOH, NH_4_Cl, NaCl, Na_2_SO_4_, NaHCO_3_, NaNO_3_, Na_2_HPO_4_·2H_2_O, and NaH_2_PO_4_·2H_2_O were purchased from Carlo Erba. H_2_O_2_ 33% w/w, HNO_3_ 70%, HClO_4_ 70%, H_3_PO_4_ 85%, NH_3_ 25%, acetic acid (glacial), acetone, isopropyl alcohol, methanol, and acetonitrile were purchased from VWR Chemicals. All reagents were used as received, and solutions prepared with ultrapure water (total organic carbon ≤ 10 ppb, resistivity ≥ 18.2 MΩ cm). Stock solutions were stored at 4 °C until use; only those from NH_2_OH and Fe­(III) were prepared daily.

A Varian Cary Scan 100 spectrophotometer was employed for colorimetric determination: (i) Cu­(I) and total Cu, adapted from Heller and Guyon^36^ employing ascorbic acid, neocuproine 1.3 M solution (dissolved in methanol), and NH_3_/NH_4_^+^ buffer pH 9.0, absorbance measured at 450 nm; (ii) Fe­(II) and total Fe, according to ISO 6332:1988, employing ascorbic acid and 1,10-phenanthroline 5 mM, dissolved in acetic acid/acetate buffer pH 4.0, absorbance measured at 510 nm; (iii) H_2_O_2_, adapted from Vojinović et al.,^37^ employing a solution containing 0.234% w/v phenol, 0.10% w/v 4-aminoantipyrine, 0.0010% w/v peroxidase, dissolved in H_2_PO_4_^–^/HPO_4_^2–^ buffer pH 7.0, absorbance measured at 505 nm.

The total organic carbon and nitrogen (TOC and TN, respectively) were measured employing a Shimadzu TOC-VCSH Total Organic Carbon Analyzer, equipped with an ASI-V autosampler and fed with zero-grade air.

Benzoate degradation was determined by HPLC-UV/vis (HPLC, YL9300 HPLC System, Lichrospher R100 RP-18 5 μm column) coupled to a UV/vis detector (λ = 235 nm) employing isocratic elution, mobile phase H_3_PO_4_ 14 mM:methanol, 60:40 (% v/v). Furfuryl alcohol (FFA) was measured employing 5% methanol and 95% H_3_PO_4_ 14 mM, λ = 220 nm. The NH_2_OH concentration was measured by its derivatization into the respective oxime with acetone 20% v/v, measuring the absorbance at 210 nm and employing the mobile phase H_2_PO_4_^–^/HPO_4_^2–^ 10 mM:acetonitrile, 97:3.^24,25^ In all cases, a flow of 1 mL min^–1^ was employed, with the column temperature fixed to 35 °C.

Benzoate 50 μM solution was stable in the presence of (separately) Cu­(II) 100 μM, H_2_O_2_ 1 mM, or NH_2_OH 250 μM for at least 4 h.

### 2.2 Experimental Procedure

BA degradation experiments were carried out for 4 h in open batch reactors containing 100 mL of the testing solution. In all cases, the experiments were performed in ultrapure water containing a fixed concentration of 50 μM BA and 100 μM Cu­(II) (except for the respective blank controls where Cu­(II) was omitted), whereas the one employed for NH_2_OH ranged from 0.05 to 1 mM. When needed, H_2_O_2_ was employed with an initial concentration of 1 mM. These conditions were selected based on similar works.^16,13,38^ Although the NH_2_OH concentration range might seem wide, it was unknown which quantity would enable the faster BA degradation, hence, its optimal value was obtained empirically.

The pH was adjusted to 3.0–9.0 with HClO_4_ or NaOH 0.1 M. Due to plausible interferences of high concentration of anions employed as buffer solutions, if required, the pH was kept constant by continuous addition of acid or base. When needed, N_2_ or O_2_ were bubbled to achieve anaerobic or oxygen-saturated conditions, respectively.

The effect of water constituents included the following: (i) chelating agents (ethylenediaminetetraacetate - EDTA) 100 μM, (ii) humic acids (10 and 100 mg L^–1^), (iii) anions (Cl^–^, NO_3_^–^, SO_4_^2–^, B­(OH)_4_^–^ - at pH 7 as H_3_BO_3_, HCO_3_^–^, and H_2_PO_4_^–^/HPO_4_^2–^) 1 mM each, and (iv) some transition metals (Fe­(III), Mn­(II), and Mo­(VI)) 100 μM each. In all cases, 1 M methanol was used to stop the reaction when taking samples at different time intervals (it was previously confirmed that this high concentration of methanol was enough to stop the oxidative reaction). When measuring the TOC-TN concentration evolution over time, the reaction was quenched by acidification until pH 2.5, as the Cu­(NH_2_OH)^2+^ complex is not formed and the reaction is stopped (*vide infra*).

The employed salts were CuSO_4_·5H_2_O, Fe­(ClO_4_)_3_·*x*H_2_O, MnSO_4_·H_2_O, and Na_2_MoO_4_·2H_2_O. The chosen concentrations of Cu­(II), NH_2_OH, H_2_O_2_, anions, and humic acids were based on similar works on the matter,^16,19,38^ whereas the concentrations of EDTA and transition metals different from Cu­(II) were 100 μM to compare their effect when added in identical stoichiometric amounts to that of Cu­(II).

After the investigation of the fundamentals behind the Cu­(II)/NH_2_OH system with experiments carried out with ultrapure water, the effectiveness of NH_2_OH in the presence of trace amounts of Cu­(II) (10 μM) toward BA degradation was studied in simulated wastewater (see composition in Table S1).

### 2.3 Cupric Ion Speciation Diagrams

When needed, Cu­(II) speciation in the presence of ligands was calculated by employing the free software HySS2009.^45^ The stability constants used in this work (e.g., log*K* of Cu­(NH_2_OH)^2+^, Cu­(HCO_3_)^+^, Cu­(HPO_4_)_(aq)_, etc.) can be found in Table S2.

### 2.4 Reactive Species Quenching and Detection

The mechanistic aspects were first investigated through competition kinetic tests by using selective scavengers in systems containing BA and NH_2_OH/H_2_O_2_, Cu­(II)/H_2_O_2_, Cu­(II)/NH_2_OH, and Cu­(II)/NH_2_OH/H_2_O_2_. The employed concentration of the selective scavengers was 100 μM each, twice the concentration of the model pollutant. Isopropyl alcohol (IPA) was used as a HO^•^ selective scavenger (*k*_IPA/HO•_ = 1.9 × 10^9^ M^–1^ s^–1^), furfuryl alcohol (FFA) as a HO^•^ and ^1^O_2_ quencher (*k*_FFA/HO•_ = 1.5 × 10^10^ M^–1^ s^–1^ and *k*_FFA/1O2_ 1.2 × 10^8^ M^–1^ s^–1^), and *p*-benzoquinone (pBQ) as a selective O_2_^•–^ scavenger (*k*_pBQ/__O__2•–_ = 9.5 × 10^8^ M^–1^ s^–1^).^39–41^ The hypothesis obtained from these experiments were confirmed by electron paramagnetic resonance spectroscopy (EPR, Bruker ESR 300E spectrometer). 18 mM 5,5-dimethylpyrroline-*N*-oxide (DMPO) or 77 mM 2,2,6,6-tetramethylpiperidine (TEMP) were employed as spin traps, being added directly to the studied process in the absence of BA and in ultrapure water. The chosen concentrations of spin traps were the same used in previous works.^38,42^

Cu­(III) detection was carried out either through EPR as well as by absorbance spectroscopy as suggested in the literature.^17,18,43^ For the first, the extended methodology suggests adding into the testing solution, besides DMPO 18 mM, methanol 10 M to observe the formation of DMPO-OCH_3_. The mechanism is based on the different reactivity that HO^•^ and Cu­(III) have with CH_3_OH and DMPO, respectively: the first reacts predominantly with CH_3_OH than with DMPO, generating ^•^CH_2_OH, consequently obtaining DMPO–CH_2_OH, whereas Cu­(III), with moderate reactivity toward CH_3_OH, reacts with DMPO, leading to DMPO^•+^, observing the adduct of DMPO-OCH_3_ in the EPR spectra.^43^ Regarding the colorimetric method, Cu­(III) forms a stable complex with IO_4_^–^ that exhibits an absorbance maximum at 415 nm. Therefore, an excess of IO_4_^–^ (20 mM) was added in the solution containing Cu­(II) 100 μM at initial pH 7 in order to detect the plausible Cu­(III) formed once NH_2_OH and/or H_2_O_2_ were added.

Regarding the detection of ^•^NO, the use of a 5 mM Fe­(II)-diethyldithiocarbamate (DETC) complex was employed as a probe to form Fe­(DETC)_2_^2+^-NO, which is active at the EPR, as suggested in other works.^24,44^

To gain complementary information to that obtained through EPR, the transformation products of DMPO and BA were analyzed by high resolution mass spectrometry (HRMS); details about the instrument and employed conditions are described in Text S1. For HRMS, DMPO 1.8 mM was employed (10 times lower than those used at the EPR) since the results were better appreciated.

The role of RNS was assessed by measuring the NO_(g)_/NO_2(g)_ released in the gaseous phase by the Cu­(II)/NH_2_OH process when increasing the BA concentration from 0 to 100 μM (in all cases, pH_0_ = 7.0, [Cu­(II)]_0_ = 100 μM and [NH_2_OH]_0_ = 250 μM). The procedure was adapted based on a previous work.^46^ A three-neck flask was filled with 100 mL of a solution of Cu­(II) 100 μM and the desired BA concentration, and each neck was closed with rubber septa precision seals (Merck). On one neck, synthetic air (N_2_:O_2_ ratio = 79:21) was flowed at 1.2 L min^–1^, and the exit gas was directed into a NO_(g)_/NO_2(g)_ detector (HORIBA APNA mod. 370) through another neck. Through the third neck, 100 μL of NH_2_OH 250 mM were added with a syringe needle to start the reaction (see the experimental setup scheme in Figure S1). The NO_(g)_ and NO_2(g)_ concentrations were registered for 1 h of process, and the moles of gas formed were calculated integrating the area under the curve of the NO_(x)_ vs time plot. Further details are given in Text S2.

### 2.5 Benzoate Oxidation by ^1^O_2_ Bimolecular Rate Constant Determination

Despite being a commonly employed model contaminant, the second order rate constant for the reaction between BA and ^1^O_2_ has never been previously reported. The details of its measurement are shown in Text S3.

### 2.6 Ecotoxicity Studies

The potential toxicity of hydroxylamine before and after reaction was tested by performing a toxicity test with freshwater microalgae*Chlorella vulgaris* (Strain CCMM 02/0205, obtained from the Marine Microalgae Culture Collection at ICMAN-CSIC; ICMAN-CCMM) maintained in enriched nutrient medium with a procedure adapted from other works.^47,48^ For microalgae tests, *C. vulgaris* cultures in the exponential growth phase were adjusted to an initial density of 10^4^ cells mL^–1^. Experiments were carried out in autoclaved glass tubes previously washed with 10% w/w HNO_3_. The following conditions were tested: (i) individual exposures to NH_2_OH 250 μM, Cu­(II) 100 μM, and BA 50 μM, and (ii) the toxicity after 4 h of Cu­(II)/NH_2_OH treatment with and without BA (all assays were performed in quadruplicate and in the presence of 1 mM phosphate buffer, pH 7.2). In all cases, 5 mL of sample were mixed with 5 mL of the culture medium (1:2 dilution factor). Tubes containing the 10 mL resulting solution were then incubated for 96 h in a climate chamber at 20 °C (±0.5 °C) under continuous illumination with photosynthetically active radiation (120 μmol photons m^–2^ s^–1^; QSL-2100 Radiometer, Biospherical Instruments Inc., USA).

After 96 h of exposure, cultures were measured by flow cytometry (Thermo Fisher Attune NxT Acoustic Focusing Cytometer), equipped with a 488 nm excitation blue laser, detectors of forward and side light scatter, and four fluorescence channels corresponding to four wavelength intervals: BL1 (530/30 nm), BL2 (574/26 nm), BL3 (695/40 nm), and BL4 (780/60 nm). End points addressed were cell density (cells mL^–1^) and the inherent cell properties (cell size, cell complexity, and autofluorescence). Forward light scatter intensity was correlated with cell size or volume, and side light scatter was correlated with intracellular complexity. Chlorophyll-a fluorescence emission (autofluorescence) was measured with the BL4 scatter. The mean values of these three parameters were given by the software in arbitrary units (a.u.). To determine and evaluate significant differences between exposures (*p* < 0.05), confidence intervals (95%) were calculated for each parameter using SPSS 15.0 software.

## 3 Results and Discussion

### 3.1 Seeking the Best Initial Concentration of NH_2_OH

The effect of initial NH_2_OH concentration for BA 50 μM degradation was first studied. In these cases, Cu­(II) concentration was 100 μM and the initial pH was adjusted to 7.0. As shown in Figure 1a, maximum efficiency was obtained with 250 μM NH_2_OH, leading to approximately 40% BA degradation in 1 h. This value is 20 times lower than the ones reported in similar works to obtain comparable BA degradation rates, which might be related to the avoidance of concentrated buffer solutions that can precipitate copper.^13,16^

**Figure 1:**
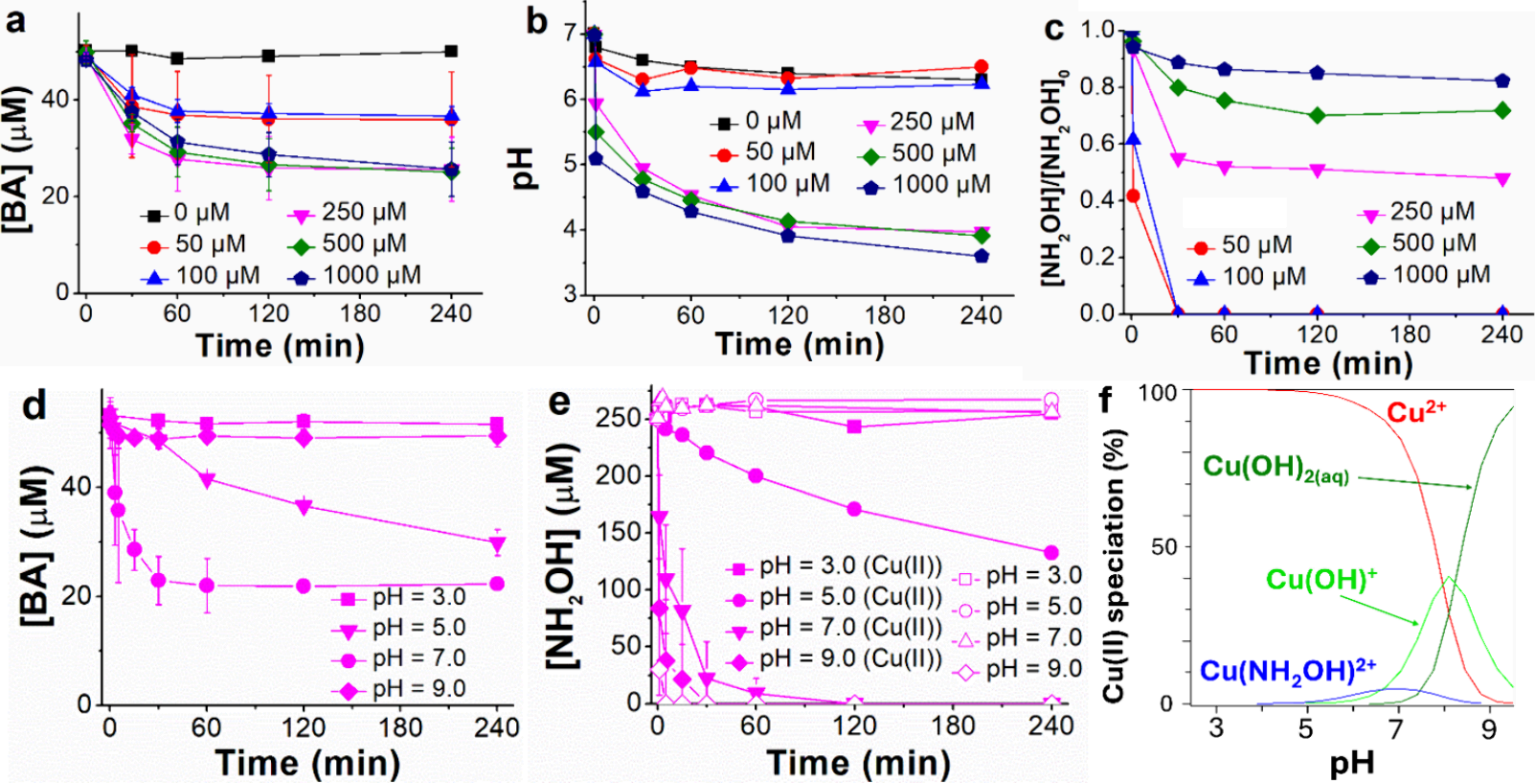
Results from the influence of NH_2_OH initial concentration and pH (the latter either associated with the NH_2_OH addition or intentionally modified) in systems containing 100 μM Cu­(II): (a–c) BA, NH_2_OH, and pH behavior with time when increasing the NH_2_OH initial concentration from 50 to 1000 μM (initial pH 7.0); (d, e) effect of fixed pH (employing continuous addition of NaOH or HClO_4_ 0.1 M) on the BA and NH_2_OH oxidation, respectively, the latter including the NH_2_OH kinetics in the absence of Cu­(II) at different pH values ([NH_2_OH]_0_ = 250 μM); (f) Cu­(II) speciation at different pH values ([NH_2_OH]_0_ = 250 μM).

At hydroxylamine concentrations of 500–1000 μM, even though it was observed that the removal kinetics were slightly slower than that with 250 μM, the BA oxidation seemed to continue descending, not observing the *plateau* obtained with the latter. Regarding the slower degradation kinetics, they were related to the acidification produced by the NH_2_OH·HCl addition, which hindered the process. As shown in Figure 1b, the pH reached values of approximately 5.0 in 1 min for NH_2_OH 1000 μM, whereas the optimal 250 μM was 6.0 in the same period. The decrease in oxidative performance by acidification was also evidenced by the lower NH_2_OH consumptions (see Figure 1c). On the other hand, regarding the absence of the BA oxidation *plateau*, it can be explained by two issues that are favored when increasing the NH_2_OH concentration: (i) the higher contribution of NH_2_OH/H_2_O_2_ parallel reaction and (ii) the higher proportion of Cu­(II) chelated by NH_2_OH at pH < 5. The roles of pH, copper complexation by NH_2_OH, and NH_2_OH/H_2_O_2_ are discussed in the following sections.

### 3.2 Role of pH: Relevance of the Cu­(NH_2_OH)^2+^ Complex in the Process Efficiency

In order to study the effect of pH without the use of buffer solutions, the solution containing BA 50 μM and Cu­(II) 100 μM was adjusted to 3.0, 5.0, 7.0, and 9.0 and remained constant after the NH_2_OH addition by continuous adjustment with NaOH 0.1 M or HClO_4_ 0.1 M as necessary. The results are shown in Figure 1d,e (BA and NH_2_OH oxidation kinetics, respectively).

The fastest BA and NH_2_OH abatements (ca. 50% BA oxidation and 90% NH_2_OH consumption in 30 min, respectively) were obtained at pH 7.0, followed by the analogous at pH 5.0 (50% decrease of BA and NH_2_OH concentrations in 4 h, respectively) and obtaining negligible BA and NH_2_OH consumptions at pH 3.0. At pH 9.0, although no BA oxidation was observed for at least 4 h, the NH_2_OH exhibited a 90% consumption in 5 min, which is in line with the NH_2_OH self-oxidation at alkaline conditions (see the obtained kinetics in the absence of Cu­(II) in Figure 1e).^49^ Therefore, the obtained BA and NH_2_OH kinetics could be perfectly explained by the proportion of Cu­(NH_2_OH)^2+^ (log *K* = 2.4, see Table S2), relevant from pH 6–8 and negligible at pH < 4 or >8.5 (see Figure 1f); the speciation of NH_2_OH in the presence of Cu­(II) as a function of pH is shown in Figure S2a.

On the other hand, TOC and TN measurements gave insights regarding the mineralization of BA and the oxidation products of NH_2_OH, respectively. Regarding the first one, the TOC remained constant for 4 h, whether the pH was kept constant at 7.0 (Figure S2b), indicating negligible BA mineralization by the Cu­(II)/NH_2_OH process. On the contrary, the TN kinetics at fixed pH 7.0 followed a similar trend to that of NH_2_OH, being negligible after 4 h of treatment (see Figure S2c), indicating that NH_2_OH is presumably oxidized into N_2_ and/or N_2_O as widely reported.^16,50^ As expected, when the experiment was carried out without controlling the pH, the TN reached a *plateau* after approximately 15 min of reaction due to the discussed acidification and lack of Cu­(NH_2_OH)^2+^ formation.

### 3.3 Competition with Other Chelating Agents: EDTA and Dissolved Organic Matter

EDTA, simulating an intentionally added chelating agent in Fenton processes, and dissolved organic matter present in natural water or wastewater were intentionally added to evaluate the effect of competing ligands with Cu­(NH_2_OH)^2+^. Cu^2+^-EDTA complexes exhibit stability constants in the 10^18^ order,^51^ inhibiting the formation of Cu­(NH_2_OH)^2+^. As expected, negligible BA oxidation was obtained in the presence of EDTA 100 μM (see Figure S3, Cu^2+^-EDTA complexes also being Fenton inactive),^7^ with a concomitant scarce NH_2_OH consumption (13% in 4 h). This was not the case for humic acids, which present low to moderate stability constants with Cu­(II), log *K* = 3–7,^52,53^ evidenced in the negligible effect of 10 mg L^–1^ content on the BA degradation (50% removal in 1 h). Only with an excess of humic acid (100 mg L^–1^) a considerable hindering effect was observed (35% BA removal in 2 h), which could also be related to the scavenging of the formed reactive species by the humic acids as widely reported for several AOPs.^38,54,55^

### 3.4 Role of Dissolved Oxygen Concentration

H_2_O_2_ is a reaction intermediate in the Cu­(II)/NH_2_OH process ([Disp-formula eqR4], [Disp-formula eqR5]). As shown in Figure 2a, the formation of H_2_O_2_ obtained its highest value at oxygen saturated conditions, accumulating almost 100 μM in 15 min, followed by its consumption due to the parallel Fenton reaction. At aerobic conditions, the H_2_O_2_ accumulation reached its maximum at 5 min with 28 μM, whereas under anaerobic conditions, the H_2_O_2_ was not detected during the whole experiment as expected. In spite of the greater H_2_O_2_ formation in the oxygen saturated system, the BA degradation was slower than that performed under aerobic conditions (40 and 50% in 4 h, respectively). This issue is in line with the fact that, in the oxygen saturated scenario, the formed Cu­(I) reacts predominantly with the O_2_ rather than with the H_2_O_2_, the Fenton reaction becoming less efficient than in the case with lower dissolved oxygen concentration, in agreement with the work of Lee et al.^16^ Finally, BA degradation under anaerobic conditions was negligible, in line with the lack of H_2_O_2_ formation.

**Figure 2:**
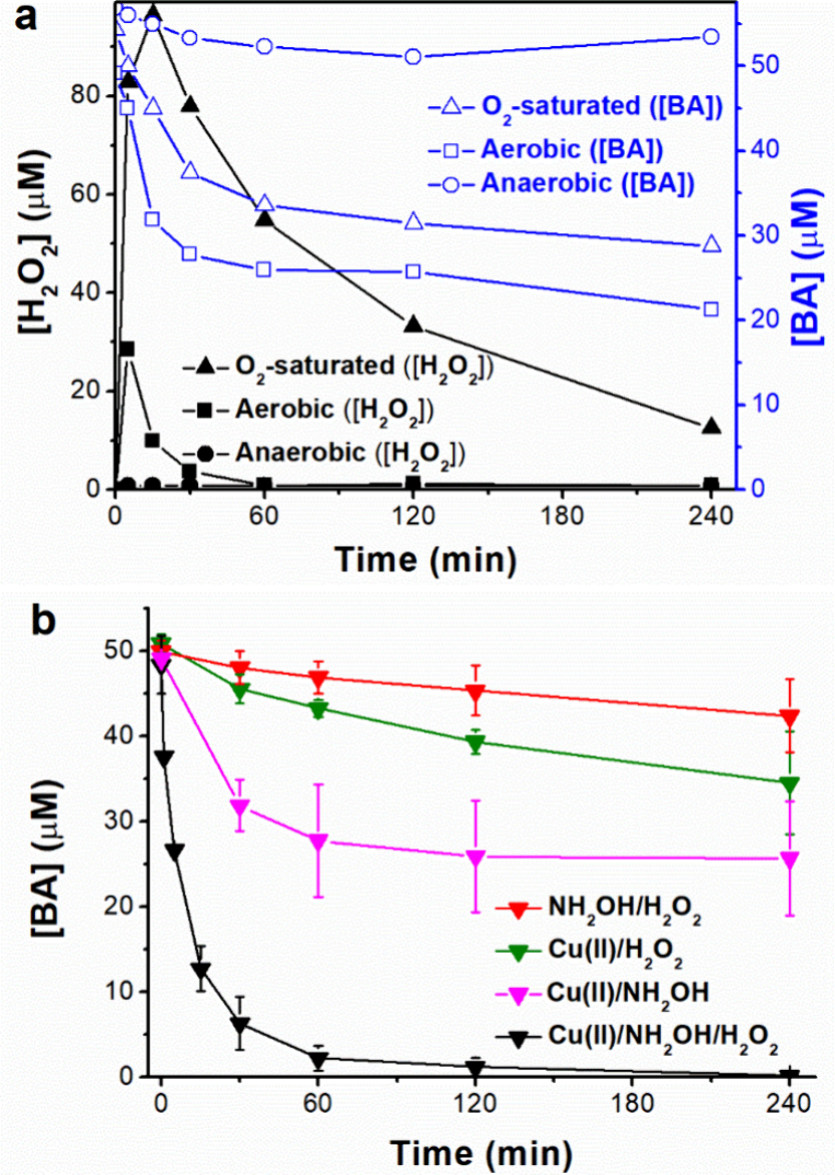
(a) BA oxidation performances obtained when changing the H_2_O_2_ formation rate (by reducing or incrementing the dissolved oxygen concentration) on the Cu­(II)/NH_2_OH reaction: H_2_O_2_ formation (left *Y*-axis, in black) and BA degradation (right *Y*-axis, in blue) at anaerobic (N_2_ bubbling), aerobic and oxygen saturated conditions (O_2_ bubbling). (b) BA oxidation performance when adding a H_2_O_2_ excess (1 mM) into the Cu­(II)/NH_2_OH process at aerobic conditions; blank experiments (NH_2_OH/H_2_O_2_ and Cu­(II)/H_2_O_2_) were also included.

### 3.5 Effect of External H_2_O_2_ Addition

Opposed to the case of O_2_-saturated conditions, in the presence of H_2_O_2_ excess (1 mM), the NH_2_OH mostly accelerates the catalytic loop by Cu­(II) reduction, acting as a cocatalyst. Therefore, the *in situ* H_2_O_2_ formation is not the rate-limiting step in the Cu­(II)/NH_2_OH/H_2_O_2_ process any longer. As shown in Figure 2b, the latter led to >80% BA degradation in 30 min (*k*_obs_ = 6.5 × 10^–2^ min^–1^), with a H_2_O_2_ consumption of approximately 25% in 4 h (see Figure S4). Regarding the blank controls (Cu­(II)/H_2_O_2_ and NH_2_OH/H_2_O_2_), they both exhibited low BA degradations (ca. 30 and 15% oxidation in 4 h, respectively), indicating a lower production of reactive species, as expected.

The respective results at fixed pH 3.0–9.0 are shown in Figure S5. NH_2_OH/H_2_O_2_ produced some degradation only at pH 3.0–5.0, being negligible at pH 7.0 or pH 9.0 (the latter also due to its low stability at alkaline conditions, as previously mentioned), in agreement with the fact that NH_3_OH^+^ has higher kinetic rate constants with H_2_O_2_ than the neutral form.^25^ The Cu­(II)/H_2_O_2_ exhibited only high BA degradation at pH 7.0, being negligible at acidic or basic conditions, whereas the fastest H_2_O_2_ consumption was at pH 9.0, in line with the literature: under alkaline conditions, in spite of the slower oxidation rates of Cu­(I) by O_2_ and the higher reactivity of copper ions toward HO_2_^–^ than H_2_O_2_, the generated reactive species are either quenched by the formed hydroxides and/or exhibit lower reactivity than those formed at acid-neutral conditions.^5,8,56^ As expected, Cu­(II)/NH_2_OH/H_2_O_2_ produced the fastest BA abatements at pH 7.0 (85% degradation in ≤1 min), although they were not complete due to the fast NH_2_OH consumption (80% oxidation in the same period). Results obtained at pH 5.0 (Figure S5f and h) are similar to those reported in Figure 2b and Figure S4, in alignment with the acidification produced by NH_2_OH addition as previously discussed. Interestingly, some BA degradation was obtained at pH 3.0 and 9.0, indicating that the cocatalytic role of NH_2_OH (even if barely interacting with Cu­(II)) helps the Cu­(II)/H_2_O_2_ to be more efficient even at acidic or alkaline conditions.

### 3.6 Effect of Anions

As shown in Figure S6, in the presence of 1 mM of the studied anions, except for HCO_3_^–^ and HPO_4_^2–^, the NO_3_^–^, Cl^–^, SO_4_^2–^, or H_3_BO_3_ did not interfere significantly with the BA degradation by the Cu­(II)/NH_2_OH process. NO_3_^–^, SO_4_^2–,^ and H_3_BO_3_ are known to be relatively inert against HO^•^, whereas the reactivity of Cl^–^ against HO^•^ at neutral pH is also very low (*k* = 10^3^ M^–1^ s^–1^).^57^ In addition, at 1 mM concentration, the plausible formation of complexes of Cu­(II) with Cl^–^, NO_3_^–^, SO_4_^2–^, or H_3_BO_3_ is low (see Table S2 to find reported stability constants). In spite of their low reactivity with HO^•^, results described in the following sections will demonstrate that the Cu­(II)/NH_2_OH process does not generate HO^•^ significantly, suggesting that these anions are also unreactive with the generated reactive species.

HCO_3_^–^ is a strong HO^•^ scavenger (*k* = 10^7^ M^–1^ s^–1^)^40^ and, at pH 7.0, forms stable complexes with Cu­(II); in the employed system, almost 60% of total copper is as CuCO_3(aq)_ (see Figure S7a). These two issues might lead to the expectation of obtaining scarce pollutant abatement by Cu­(II)/NH_2_OH in the presence of 1 mM HCO_3_^–^. However, the observed degradation of BA at the first 5 min was even faster than in the absence of anions, with 30% removal (equivalent to a *k*_obs_ = 1.7 × 10^–2^ min^–1^). An analogous trend was observed for the NH_2_OH, with a consumption >95% in the same period, explaining the subsequent hindering on the BA oxidation. In part, these results can be justified by the buffering effect produced by the HCO_3_^–^/CO_2(aq)_ couple (p*K*_a_ = 6.4), which avoids the system acidification produced by the addition of NH_2_OH addition. On the other hand, HCO_3_^–^ should catalyze the NH_2_OH oxidation either by Cu­(II) or dissolved oxygen. Regarding the first case, it was reported that CuCO_3(aq)_ accelerate the cupric ion Fenton cycle,^7,58^ which might reasonably explain the observed phenomena. Regarding the second possibility, the stability of NH_2_OH 250 μM solution in the presence of 1 mM HCO_3_^–^ was verified, observing that it remained stable for at least 1 h under stirring, suggesting that the main NH_2_OH decomposition enhancement occurs due to the CuCO_3(aq)_/H_2_O_2_ reaction.

The final exception concerning the explored anions were the phosphates. Different from HCO_3_^–^, H_2_PO_4_^–^/HPO_4_^2–^ have a lower reactivity toward HO^•^ (*k* = 1 × 10^4^–1 × 10^5^ M^–1^ s^–1^)^40^ and form complexes with Cu­(II) with lower stability constants (see Table S2). In fact, under the employed conditions, the Cu­(II) speciation diagram does not differ from that in the absence of phosphates (compare Figure 1f with Figure S7b). Therefore, the obtained results (70% BA oxidation in 4 h and >95% NH_2_OH consumption in 2 h) are reasonably explained just by the buffering effect of H_2_PO_4_^–^/HPO_4_^2–^ (p*K*_a_ = 7.2), which are comparable to that obtained in ultrapure water at fixed pH 7.0 with continuous addition of NaOH (Figure 1d,e).

### 3.7 Effect of Transition Metals

Fe­(III), Mn­(II), and Mo­(VI) are transition metals commonly found in natural water with the potential to drive their parallel Fenton-like reactions.^28–32^ As shown in Figure 3a,b, the presence of 100 μM Fe­(III) or Mo­(VI) slowed down the BA degradation by the Cu­(II)/NH_2_OH process because they accelerated the NH_2_OH consumption (a 90% consumption in 4 h in the presence of the Fe­(III) or Mo­(VI) was observed, being 60% by cupric ions alone), competing for it with Cu­(II). Since Fe­(III) and Mo­(VI) corresponding Fenton reactions are inefficient in the working conditions (*vide infra*), the BA abatement hindering in the presence of the aforementioned is then logical. Finally, with the addition of 100 μM Mn­(II), a negligible effect was observed because Mn­(II) exhibits a scarce catalytic effect on H_2_O_2_ decomposition, and low Mn­(III) (that could eventually be reduced by the NH_2_OH) should be formed too.^8,32^

**Figure 3:**
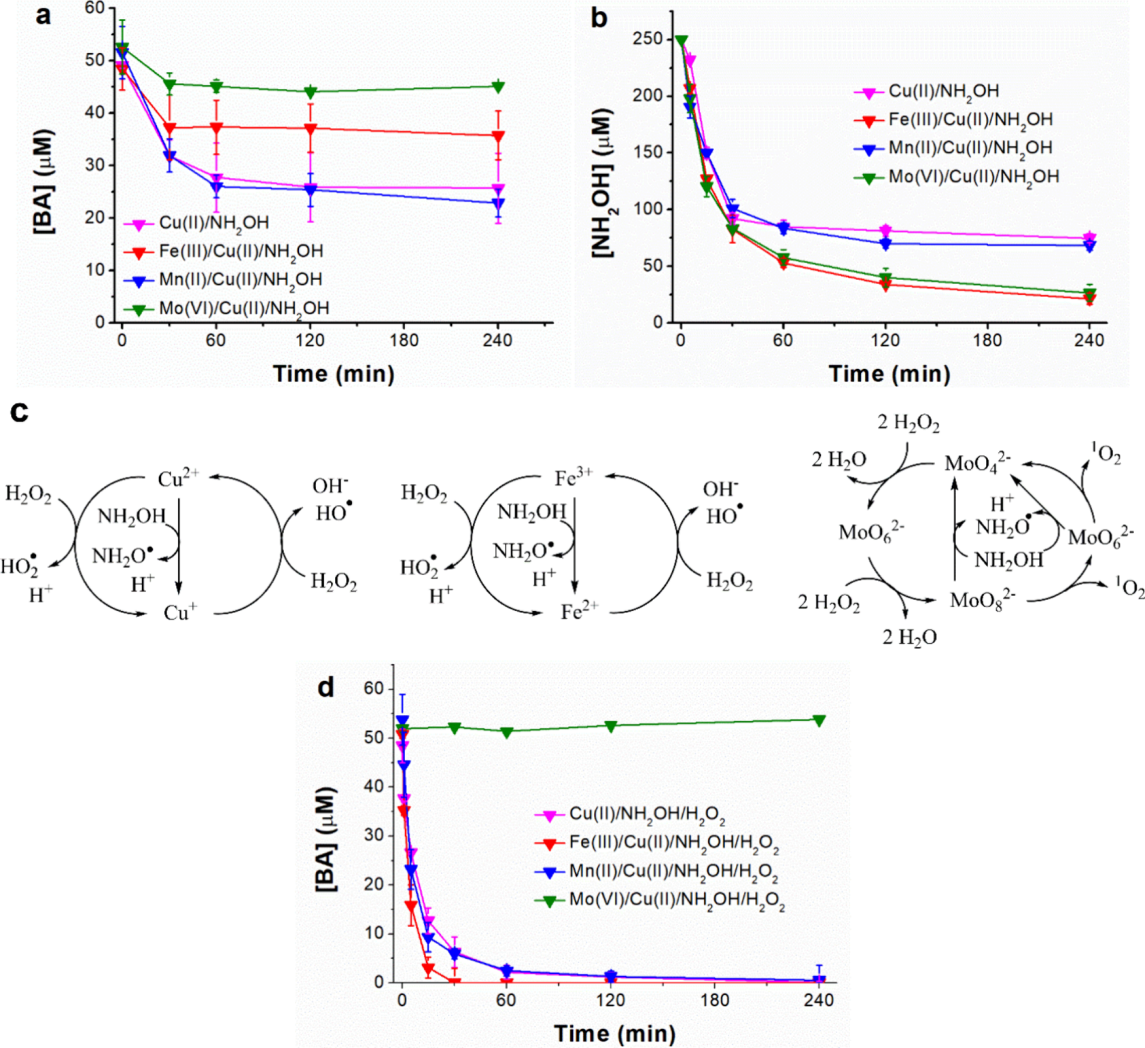
Results obtained from the addition of Fe­(III), Mn­(II), or Mo­(VI) into the Cu­(II)/NH_2_OH or Cu­(II)/NH_2_OH/H_2_O_2_ processes: (a) BA and (b) NH_2_OH kinetics, respectively, affected by the addition of Fe­(III), Mn­(II) or Mo­(VI) in the Cu­(II)/NH_2_OH process; (c) involved reactions of NH_2_OH and H_2_O_2_ with Cu­(II), Fe­(III), and Mo­(VI); (d) BA oxidations by Cu­(II)/NH_2_OH/H_2_O_2_ with Fe­(III), Mn­(II),, or Mo­(VI). Conditions: pH_0_ = 7.0; [Cu­(II)]_0_ = 100 μM, [Fe­(III)]_0_ = 100 μM, [Mn­(II)]_0_ = 100 μM, [Mo­(VI)]_0_ = 100 μM, [NH_2_OH]_0_ = 250 μM, and [H_2_O_2_]_0_ = 1 mM.

To gain further insights into the hindering effect of the Fe­(III) and Mo­(VI), the BA removals were evaluated by the addition of H_2_O_2_ 1 mM (alone and with NH_2_OH 250 μM), without Cu­(II). As shown in Figure S8a, nondetectable BA degradations were observed either by Fe­(III)/H_2_O_2_ or Mo­(VI)/H_2_O_2_, indicating that the parallel contribution toward pollutant removal of the respective Fenton reactions is null. Nevertheless, the H_2_O_2_ consumption for Mo­(VI) was considerable, observing 43% decrease in 4 h, whereas for Fe­(III), it was 18% in the same period. In the case of Fe­(III)/H_2_O_2_ reaction, these results were expected, as it is widely known that its catalytic effect at circumneutral pH conditions is low due to the precipitation of iron;^6^ measurements of the dissolved iron content at the beginning of the experiment resulted in 60 μM, being of barely 20 μM at the end of the process. Regarding Mo­(VI)/H_2_O_2_, it can be assumed that, although the catalytic decomposition of H_2_O_2_ was more efficient than that of Fe­(III), the formed reactive species (e.g., ^1^O_2_ and Mo­(VI)-peroxo species)^59,60^ should be produced in low quantities and/or cannot oxidize the BA considerably.

When combining H_2_O_2_ and NH_2_OH, fast BA oxidations were observed in the case of Fe­(III) (>99% in 1 h, see Figure S8b), in agreement with other works mentioning that the cocatalytic effect of NH_2_OH can help to drive efficient iron-based Fenton reactions even at circumneutral pH values without the addition of chelating agents.^6,61,62^ On the contrary, the BA oxidation was still negligible for Mo­(VI)/NH_2_OH/H_2_O_2_, even though an outstandingly fast consumption of NH_2_OH and H_2_O_2_ was observed, exhibiting >99% and 75% abatement in 15 min, respectively (see Figure S8c). Therefore, molybdenum ions catalyze the decomposition of NH_2_OH and H_2_O_2_ faster than the other transition metals. In fact, different from Fe­(III) or Cu­(II), it was verified that Mo­(VI) was not able to oxidize NH_2_OH by its own (negligible concentration decay in 4 h when combining 250 μM NH_2_OH with 100 μM Mo­(VI)), suggesting that NH_2_OH should rapidly reduce Mo­(VI)-peroxo species formed during the Mo­(VI)/H_2_O_2_ process back into MoO_4_^2–^, accelerating the catalytic cycle. These reactions are summarized in Figure 3c.

Regarding the Mn­(II)/H_2_O_2_ reaction, negligible BA abatement was observed; when employing Mn­(II)/NH_2_OH/H_2_O_2_, ca. 20% BA oxidation in 4 h was obtained, which is comparable to that with NH_2_OH/H_2_O_2_ as expected. Results are shown in Figure S8a and b, respectively.

With this background, once the transition metals were added to the Cu­(II)/NH_2_OH/H_2_O_2_ process, the expected trend was observed: Fe­(III) produced a slight enhancement of the oxidative process, Mo­(VI) hindered it completely, and Mn­(II) was inert (Figure 3d). Although some authors reported a positive contribution of Mo­(VI) to pollutant abatement in similar systems,^29^ these results clearly indicate that MoO_4_^2–^ is an interference in the Cu­(II)/NH_2_OH/H_2_O_2_ process that leaves the system with scarce levels of NH_2_OH and H_2_O_2_, reducing the efficiency of the Cu­(II)/NH_2_OH/H_2_O_2_ process and, in exchange, the Mo­(VI)/H_2_O_2_ reaction generating reactive species with low reactivity towards BA.

### 3.8 Effect of Selective Scavenger Addition

The BA degradation profiles shown in Figure S9a suggested that ^1^O_2_ should be the ROS responsible for BA oxidation, as the FFA was the scavenger producing the greatest hindering effect (only 6% oxidation in 4 h), IPA generating a slight oxidation rate reduction (40% BA removal in 1 h), being negligible with pBQ (50% removal in 1 h). The insignificant role of HO_2_^•^/O_2_^•–^ was expected, as it rapidly reacts with Cu­(I, II) ions (*k* ≈ 10^9^ M^–1^ s^–1^).^16,39^ Although more exacerbated, similar results with the external addition of H_2_O_2_ 1 mM were observed (Figure S9b), with the controls of Cu­(II)/H_2_O_2_ and NH_2_OH/H_2_O_2_ being equally affected by the three scavengers (Figure S9c and d).

Even though it might be reasonable to observe BA degradation by HO^•^, the role of ^1^O_2_ was unclear, as BA has a very low second-order kinetic constant with ^1^O_2_, estimated to be <5 × 10^5^ M^–1^ s^–1^ (see Text S3), in line with the reported bimolecular oxidation constants of ^1^O_2_ with similar compounds previously reported.^63^ Furthermore, the ^1^O_2_ production by Cu­(II)/NH_2_OH seemed negligible (see FFA degradation kinetics with and without excess IPA in Figure S10). These results are also in agreement with the scarce BA degradation observed for the Mo­(VI)/H_2_O_2_ process, where ^1^O_2_ is the ROS formed in the respective Fenton mechanism.^59,60^ Therefore, the higher quenching produced by FFA is most likely related to its higher reactivity with HO^•^ than that of IPA (*k*_FFA/HO•_ = 1.5 × 10^10^ M^–1^ s^–1^, whereas *k*_IPA/HO•_ = 1.9 × 10^9^ M^–1^ s^–1^).^39–41^

### 3.9 Electron Paramagnetic Resonance Measurements

When performing the EPR analysis of the reaction in the presence of TEMP 77 mM, clear signals of the TEMP-O spin adduct were observed in all cases (1:1:1 triplet with a hyperfine separation constant, α^N^ = 16 G, see Figure S11), suggesting the formation of ^1^O_2_, which is in agreement with some works employing AOPs based on Cu.^18,22^ In a similar system, Song et al. proposed that Cu­(II)/ascorbic acid did not follow a Haber–Weiss mechanism to form ^1^O_2_, being generated from a Cu­(I)-ascorbic acid-H_2_O_2_ intermediate.^21^ However, TEMP-O false-detection was previously reported in ^1^O_2_-absent systems,^64^ indicating that its observation by EPR does not unequivocally mean the presence of this ROS. Based on the previously given discussion, our results indicated that ^1^O_2_ formation was negligible (or, in any case, non-important for the BA degradation); hence, the TEMP oxidation into TEMP-O should not be linked to the latter, being a false-detection as reported in other works.

Regarding HO^•^, its detection was carried out by employing the spin trap, DMPO. As expected, the DMPO–OH characteristic signal (α^H^ = α^N^ = 14.9 G, with 1:2:2:1 intensity ratio) was observed for Cu­(II)/H_2_O_2_ and NH_2_OH/H_2_O_2_ (see Figure 4a), in line with the literature.^18,25^ Interestingly, noise was observed when combining Cu­(II) with NH_2_OH (with or without H_2_O_2_), in agreement with the work by Lee et al.^16^ Noteworthy, these authors also reported the absence of DMPO–OH by Cu­(II)/H_2_O_2_, which is strange at a certain point and being the only difference observed when comparing both works.

**Figure 4:**
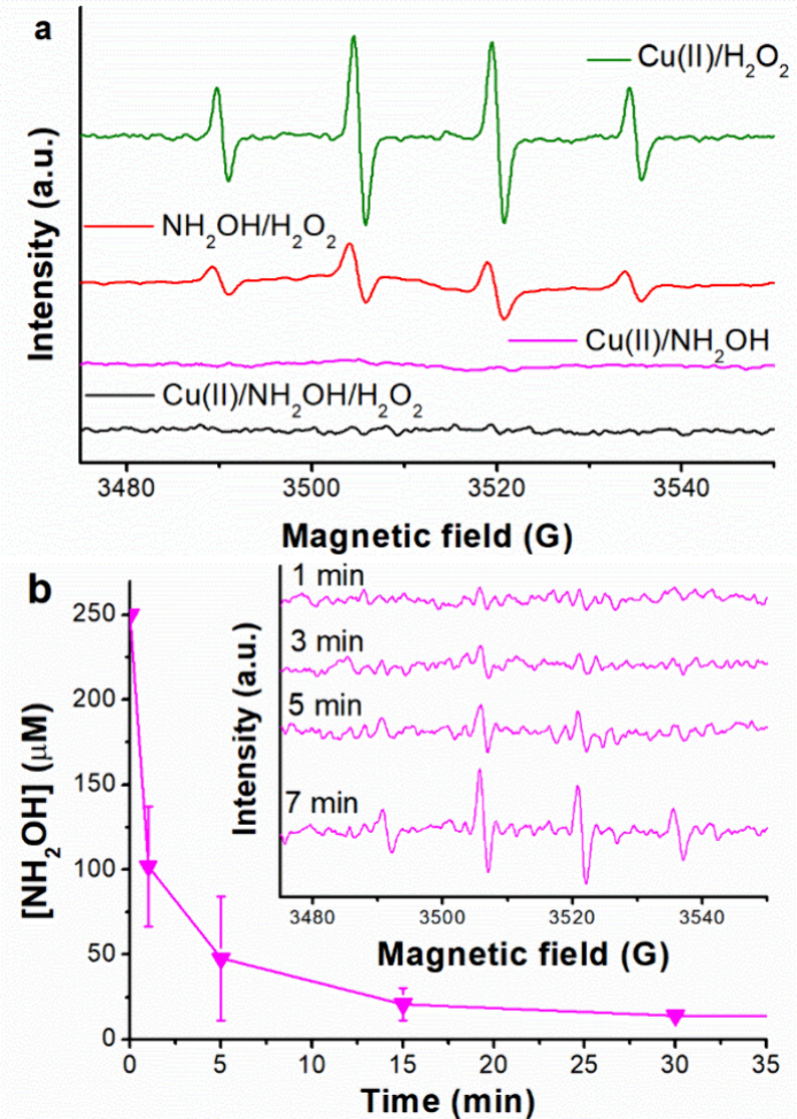
Results related to the reactive species detection: (a) EPR spectra obtained after 5 min of reaction by the different studied processes in the presence of DMPO 18 mM; (b) detection of the DMPO–OH signal once NH_2_OH is consumed during the Cu­(II)/NH_2_OH process in the presence of HCO_3_^–^ 1 mM, EPR taken at different time intervals and corresponding NH_2_OH kinetics shown.

Since the DMPO concentration was 18 mM (72 times higher than that of NH_2_OH), it is unlikely to explain the absence of DMPO–OH due to the HO^•^ fast scavenging by NH_2_OH (*k* = 9.5 × 10^9^ M^–1^ s^–1^).^65^ Therefore, the presence of NH_2_OH should, in a way, avoid the formation of HO^•^ by the Cu-Fenton reaction. To confirm this hypothesis, EPR spectra were recorded in different time intervals during the reaction of Cu­(II)/NH_2_OH in HCO_3_^–^ at 1 mM, i.e., under conditions where the NH_2_OH is rapidly consumed (see Figure S6b). As expected, the DMPO–OH was detected once the NH_2_OH concentration was negligible due to the reaction between copper ions and residual H_2_O_2_ (see Figure 4b).

Based on the above-mentioned statements, the BA observed degradation by the Cu­(II)/NH_2_OH­(/H_2_O_2_) processes cannot be due to (principally) the HO^•^, remaining two possibilities that could coexist: Cu­(III) or reactive nitrogen species (RNS, e.g., NH_2_O^•^, ^•^NO).

To verify if Cu­(III) was formed, we tried with the detection of DMPO-OCH_3_ by EPR (methodology described in Section 2.4), where, besides DMPO 18 mM, CH_3_OH 10 M was also added. As shown in Figure S12, the spectra were very low in intensity and, most importantly, more related to DMPO–CH_2_OH (α^N^ = 15.7 G and α_β_^H^ = 22.4 G) rather than DMPO-OCH_3_ (α^N^ = 14.4 G, αγ^H^ = 1.3 G, and α_β_^H^ = 10.2 G). Regarding the RNS, no shreds of evidence of NH_2_O^•^ or ^•^NHOH (both EPR-active)^25,66^ nor ^•^NO (usually indirectly detected by the formation of oxidized DMPO, DMPOX, observing an adduct with α^N^ = 7.2 G, αγ^H^ = αγ’^H^ = 4.1 G)^18,67^ were observed. The use of a 5 mM Fe­(DETC)_2_^2+^ complex was also employed as a typical probe of ^•^NO, but the characteristic signals of Fe­(DETC)_2_^2+^-NO (with a triplet α^N^ = 13.5 G) were not detected. However, studies employing this probe have either used NH_2_OH to activate peroxides without involving transition metals,^24^ or relied exclusively on iron as the catalyst.^44^ In this system, the presence of Cu­(I, II) could easily compete with Fe­(II) toward the DETC chelation, being a plausible interference that might hinder the Fe­(DETC)_2_^2+^-NO formation.

### 3.10 Trivalent Copper Detection by the Colorimetric Method with Periodate

The detection of the Cu­(III)-periodate complex by spectrophotometry (absorbance at 415 nm) was also evaluated. IO_4_^–^ 20 mM was added into the solution containing 100 μM Cu­(II) at initial pH 7.0, shortly before adding 250 μM NH_2_OH. No absorbance band was observed in the range of 380–430 nm for the whole duration of the experiment. The same result was obtained when H_2_O_2_ 1 mM was added or the pH was fixed to 7.0 (by constant NaOH 0.1 M addition or in the presence of HPO_4_^2–^ 1 mM). These results suggest that either the reported methods (the colorimetric and the previously discussed formation of DMPO-OCH_3_, respectively) are not robust, or the Cu­(III) lifetime (supposing it is formed) is very short, eventually quenched by NH_2_OH or H_2_O to form Cu­(II), not being detected, as reported in other works.^19,68^

### 3.11 High-Resolution Mass Spectrometry Analysis

HRMS measurements were carried out to elucidate the EPR-silent transformation products of DMPO obtained by Cu­(II)/NH_2_OH, with and without H_2_O_2_. Results were compared with those obtained by Cu­(II)/H_2_O_2_ and NH_2_OH/H_2_O_2_ (HO^•^-based AOP). These results are shown in Figure S13 and Table S3.

High intensity signals of [DMPOH–OH]^+^ (*m*/*z* = 130.0863) were observed in the HRMS analysis from the samples of Cu­(II)/H_2_O_2_ and NH_2_OH/H_2_O_2_ with DMPO 1.8 mM as well as *m*/*z* = 128.0706 (DMPOHX), which were also recognizable, in line with other works observing the aforementioned signals when studying reactions generating HO^•^ to oxidize DMPO.^69–71^ The high intensity signals corresponding to *m*/*z* = 136.0733 and 227.1755 were observed in the standard solution of DMPO and correspond to [DMPO^23^Na]^+^ and [DMPO–DMPO]^+^ (the dimer), respectively, which are also reported to be observed in DMPO solutions by HRMS.^71^ On the other hand, the high intensity signals at *m*/*z* = 121.9661 and 144.9821 were observed when adding Cu­(II) into the DMPO solution, which are related to copper complexes with impurities from the DMPO solution or from the instrument itself (the 144.9821 signal is a typical signal obtained by [Cu­(CH_3_CN)_2_]^+^).^72^

In the cases of Cu­(II)/NH_2_OH, with or without H_2_O_2_, the presence of HO^•^ was also observed by the detection of [DMPOH–OH]^+^, although with a lower intensity compared to that with Cu­(II)/H_2_O_2_ or NH_2_OH/H_2_O_2_, in line with the EPR results. Although RNS could also form DMPO–OH by the H-abstraction mechanism,^73^ nitrogenated-DMPO byproducts were not detected. Noteworthy, nitro-derivatives were observed when analyzing the solution coming from the BA oxidation by Cu­(II)/NH_2_OH (see Figure S14 and Table S4), in agreement with the oxidation byproducts reported by Lee et al.,^16^ confirming an active role of RNS. Therefore, the hydroxylated byproducts produced by the Cu­(II)/NH_2_OH process should be formed by H-abstraction by RNS, followed by the addition of water molecules. A tentative degradation pathway for BA is shown in Figure S15.

Finally, to consolidate the hypothesis of scarce Cu­(III) formation, in a similar way to the one performed with EPR, samples from the Cu­(II)/NH_2_OH/H_2_O_2_ process in the presence of DMPO 1.8 mM and CH_3_OH 10 M were measured. A low-intensity signal at *m*/*z* = 144.1020 was observed (see Figure S13), corresponding to either DMPO-OCH_3_ or DMPO–CH_2_OH. Noteworthy, in line with EPR results, the fragmentation analysis of this adduct (Figure S16) did not give clear information if it was DMPO–CH_2_OH, DMPO-OCH_3_, or a mixture of both.

### 3.12 NO and NO_2_ Formation Measurements

Since the EPR and HRMS measurements did not allow an unequivocal confirmation of the predominant role of RNS over HO^•^ or Cu­(III), the NO_(g)_ and NO_2(g)_ formation by the reaction of Cu­(II)/NH_2_OH in the presence of increasing BA concentrations was measured (in the gaseous phase) as described in Section 2.4. As shown in Figure 5, the NO_(g)_ formation was proportional to the BA concentration within the range of 0–50 μM, and the NO_2(g)_ concentration was negligible in all cases; for 100 μM BA, the ^•^NO formation seems to reach a maximum.

**Figure 5:**
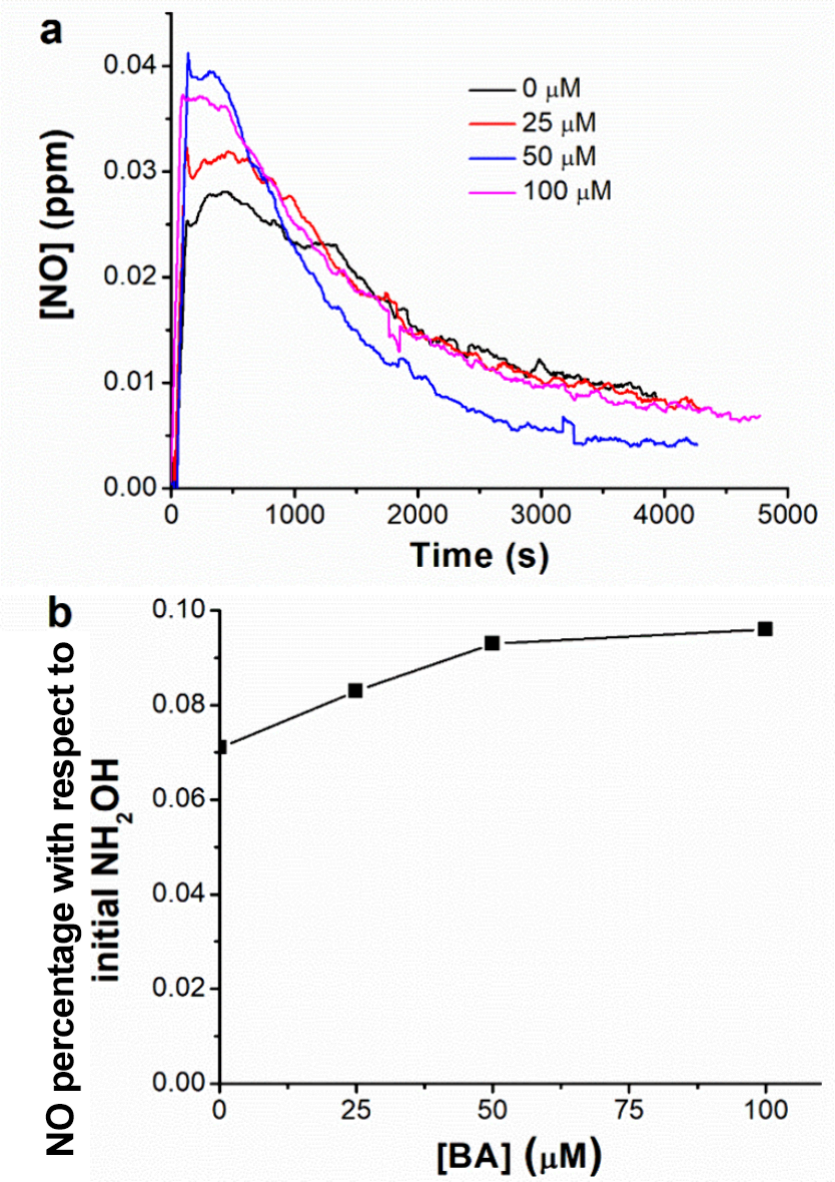
NO released in the gaseous phase during the Cu­(II)/NH_2_OH process with increasing BA concentrations (from 0 to 100 μM): (a) NO concentration over time; (b) NO formation percentage with respect to the initial NH_2_OH content (integration performed during the first 15 min of reaction, before reaching the *plateau* due to the discussed acidification). In all cases, pH_0_ = 7.0; [Cu­(II)]_0_ = 100 μM, and [NH_2_OH]_0_ = 250 μM. NO_2_ concentration was always below the detection limit.

On the one hand, the fact that NO_(g)_ (or ^•^NO) was detected confirms that it might eventually react with BA (explaining the observed maximum), as hypothesized in the previous sections. On the other hand, since ^•^NO reacts with NH_2_O^•^ ([Disp-formula eqR6]),^74^ the observed correlation between [^•^NO] and [BA] can be reasonably explained by the fact that the model contaminant should react with NH_2_O^•^ ([Disp-formula eqR7]) even faster than with ^•^NO, observing a higher concentration of the latter when increasing that of BA. These results are in line with our previous assumptions stating that RNS have a predominant role in the Cu­(II)/NH_2_OH process with respect to HO^•^ or Cu­(III).

NO•+NH2O•→N2O+H2O R6

$$\mathrm{BA}+{\mathrm{NH}}_{2}O^{•}\to \mathrm{products}$$ R7

### 3.13 Toxicity Assays

Although the high toxicity of NH_2_OH is well-known,^75^ most works employing this reagent in AOPs for water treatment did not report the ecotoxicity measurements.^16,13,24^ Therefore, we performed the Cu­(II)/NH_2_OH process with and without BA, and we compared the ecotoxicity with the individual reagents (Cu­(II), NH_2_OH, and BA, as well as the control). These experiments were carried out in the presence of 1 mM H_2_PO_4_^–^/HPO_4_^2–^ buffer to avoid toxicity contribution of acidification and ensure a >95% consumption of the NH_2_OH after 4 h of treatment (see discussion about the effect of phosphates in Section 3.6).

Overall, the data shown in Figure 6 indicate that NH_2_OH alone induces the strongest toxicity, marked by low cell survival, loss of chlorophyll autofluorescence, and morphological damage.^47,48^ The Cu-driven reactions reduce this impact, yielding higher survival and less pronounced structural and photosynthetic impairment. Nevertheless, although the Cu­(II)/NH_2_OH system showed significantly reduced toxicity compared to NH_2_OH alone, as evidenced by differences in cell density and autofluorescence measurements, it was higher than that of Cu­(II), suggesting that trace concentrations of NH_2_OH remained in the final effluent. The toxicity from nitrobenzoates should be negligible in comparison to that of residual NH_2_OH, since the results obtained by the Cu­(II)/NH_2_OH process with or without the BA were similar.

**Figure 6:**
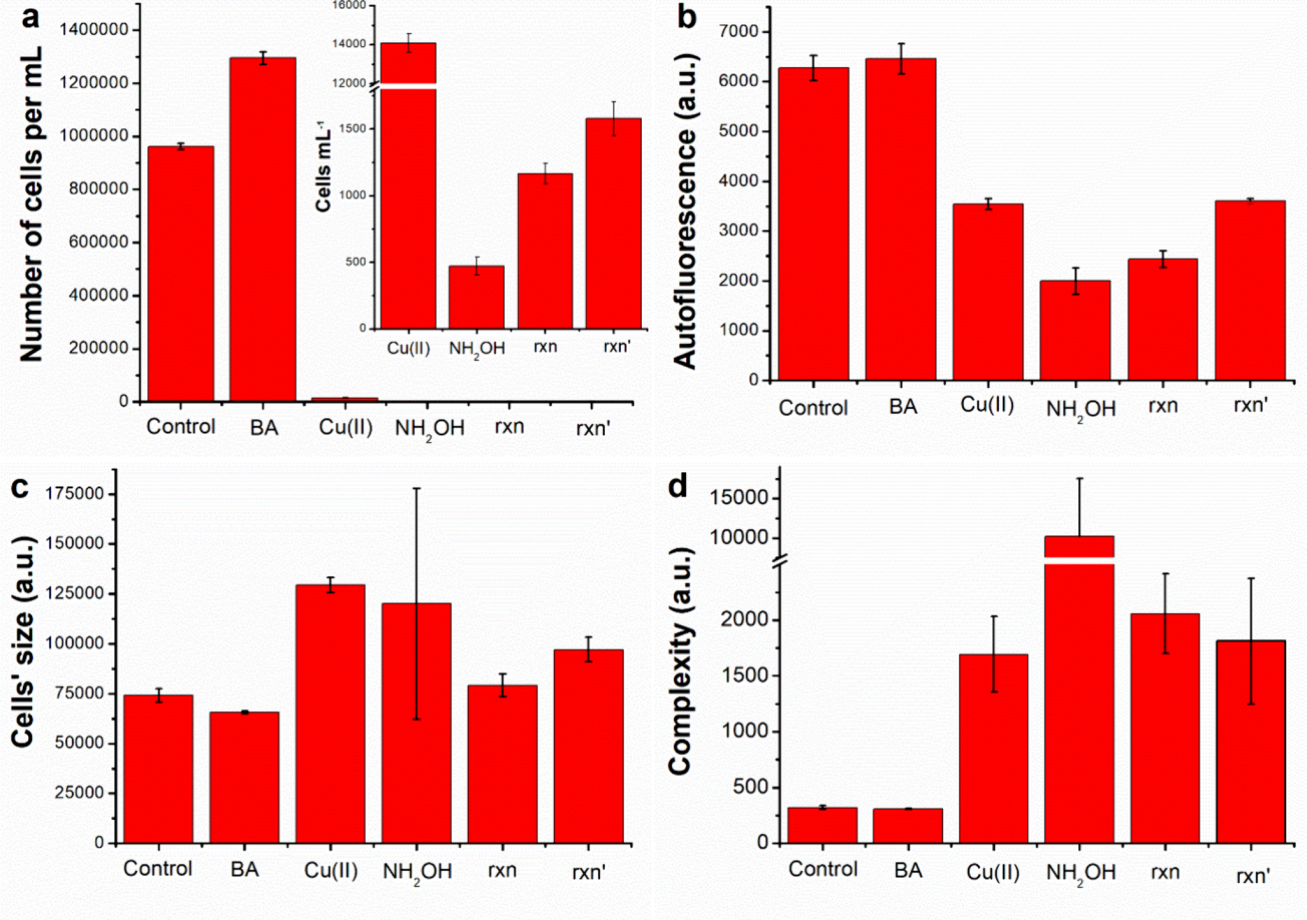
Ecotoxicity results of Cu­(II), NH_2_OH, BA, Cu­(II)/NH_2_OH (rxn), and Cu­(II)/NH_2_OH/BA (rxn’) after 96 h of exposure (dilution 1:2): (a) cell density (results from Cu­(II), NH_2_OH, rxn, and rxn’ augmented as the inset for better visualization), (b) autofluorescence, (c) cells’ size, and (d) cells’ complexity. In all of the studied solutions, H_2_PO_4_^–^/HPO_4_^2–^ 1 mM was added; “control” refers to the toxicity of the phosphate buffer alone.

### 3.14 Performance in Simulated Wastewater

To verify the usefulness of the studied process in a realistic scenario, BA 50 μM degradations were performed in simulated wastewater (composition shown in Table S1) containing trace amounts of Cu­(II) (10 μM). As shown in Figure S17a, the single addition of NH_2_OH 250 μM did not produce considerable pollutant abatement, related to the fact that with 10 μM Cu­(II), the *in situ* Fenton-like process seems to be inefficient in a realistic scenario (BA removals <10% in 4 h). The NH_2_OH was not the limiting reagent, as when adding 2500 μM of it (taking advantage of the buffered pH = 7.8), the pollutant removals did not improve. The reason was the presence of trace amounts of Cu­(II), which were not sufficient to obtain reasonable degradation rates even when combining NH_2_OH with H_2_O_2_ (17 ± 5% in 4 h). Only when incorporating additional Cu­(II) (100 μM as final concentration) and 2500 μM NH_2_OH fast BA degradations were observed, 52 ± 2% in 4 h (*k*_obs_ = 2.5 × 10^–3^ min^–1^), increasing to 68 ± 1% in 4 h (*k*_obs_ = 9.1 × 10^–3^ min^–1^) in the presence of H_2_O_2_ 1 mM. However, in neither of these cases considerable BA mineralization was observed after 4 h, in line with the results shown in Figure S2b and the obtained slow removal rates.

Finally, since the role of RNS was relevant within the studied cases, TN measurements were carried out to discard plausible formation of nitrogenated intermediates. As shown in Figure S17b, the TN corresponding to NH_2_OH is rapidly consumed during the first 30 min until reaching ca. 40 mg L^–1^, which is the equivalent of the 3 mM NO_3_^–^ initially present in the simulated wastewater.

## 4 Conclusions

The obtained results suggest that the NH_2_OH should affect the Fenton mechanism of Cu­(I, II)/H_2_O_2_, with HO^•^ or Cu­(III) not being significantly formed. A plausible explanation could be that a fraction of Cu­(I) might be chelated by NH_2_OH and both complexes, Cu­(NH_2_OH)^2+^ and Cu­(NH_2_OH)^+^, should react with H_2_O_2_ faster than Cu­(II) and Cu­(I), respectively, generating RNS as reactive species. These RNS could also explain the reasonably good performance observed in the presence of 1 mM HCO_3_^–^ (a considerable interference in most AOPs due to the significant scavenging of HO^•^). Further work is required to elucidate the Fenton mechanism of Cu­(I, II)/H_2_O_2_ in the presence of NH_2_OH, putting a special focus on the RNS, which is usually overlooked. On the other hand, ecotoxicity analysis demonstrated that even though >95% of NH_2_OH was consumed during the process of Cu­(II)/NH_2_OH at pH 7 and this one undergoes into gaseous byproducts, trace NH_2_OH levels should remain, as the ecotoxicity analysis still demonstrated considerable cellular damage.

On the other hand, although the idea of taking advantage of the naturally present Cu­(II) in some systems (usually in concentrations of 1–20 μM) to produce an *in situ* Fenton-like reaction by single addition of a reducing agent (in this work, NH_2_OH) has been stated in the literature as a promising wastewater treatment, low performance on simulated wastewater was observed. Only with concentrations of Cu­(II) ca. 100 μM (= 6.3 mg L^–1^) the treatment had a considerable degree of efficiency. However, the needed Cu­(II) concentrations to be effective are not so high, with some water directives allowing copper concentrations in the range of 1–2 mg L^–1^ for drinking water. Consequently, copper concentrations in the order of 1–10 mg L^–1^ could be reasonably proposed only for water treatments where the effluent regulation allows such levels. In these cases, one must bear in mind specific interferences such as Mo­(VI) (whose peroxo-complexes rapidly consume the NH_2_OH even in the presence of trace H_2_O_2_ concentrations) or Fe­(III) (which competes for the NH_2_OH consumption with Cu­(II), although this could be solved by adding higher NH_2_OH concentrations if the external addition of H_2_O_2_ wants to be avoided). Chelating agents that compete for copper chelation will hinder Cu­(NH_2_OH)^2+^ formation, reducing the efficiency of the AOP.

## Supplementary Material



## Notes

Text S1 (Orbitrap analysis conditions), Text S2 (calculation of NO moles formation details), Text S3 (benzoate-^1^O_2_ bimolecular rate constant determination), Table S1 (composition of simulated wastewater), Table S2 (equilibrium constants, *K*_a_, *K*_f_, *K*_sp_, of diverse compounds), Tables S3 and S4 (detected compounds by HRMS of DMPO and BA, respectively), Figure S1 (NO and NO_2_ detection setup scheme), Figure S2 (NH_2_OH speciation in the presence of Cu­(II) and total nitrogen kinetics at fixed pH = 7, and TOC and TN results), Figure S3 (effect of EDTA and humic acids on BA oxidation), Figure S4 (H_2_O_2_ consumption by Cu­(II)/NH_2_OH/H_2_O_2_ process at initial pH 7), Figure S5 (BA, NH_2_OH, and H_2_O_2_ kinetics by NH_2_OH/H_2_O_2_, Cu­(II)/H_2_O_2_, and Cu­(II)/NH_2_OH/H_2_O_2_), Figure S6 (BA and NH_2_OH kinetics by Cu­(II)/NH_2_OH in the presence of different anions), Figure S7 (Cu­(II) speciation with pH in the presence of HCO_3_^–^ and HPO_4_^2–^ 1 mM, respectively), Figure S8 (complementary results obtained by the use of Fe­(III), Mn­(II), and Mo­(VI)), Figure S9 (effect of selective scavengers, FFA, IPA, and pBQ), Figure S10 (FFA degradation by the Cu­(II)/NH_2_OH process with and without IPA 10 mM), Figure S11 (TEMP-O detection at the EPR), Figure S12 (test of Cu­(III) detection in the EPR by addition of DMPO and CH_3_OH), Figure S13 (HRMS spectrograms of DMPO), Figure S14 (HRMS spectrogram from BA oxidation by Cu­(II)/NH_2_OH), Figure S15 (benzoate degradation pathway), Figure S16 (fragmentation signals of DMPO-OCH_3_/DMPO–CH_2_OH), and Figure S17 (BA degradation kinetics in simulated wastewater and TN behavior) (PDF)
