# Supplementary material for: New Insights into the In Situ Fenton-like Process by Copper(II)-hydroxylamine Coupling: Reactive Species, Applications, and Limitations
Source: ACS ES T Water. 2026 Jan 27;6(2):817–29. doi: 10.1021/acsestwater.5c00913 (PMC12910991; doi:10.1021/acsestwater.5c00913)
Supplement: Supplementary file 1 [file ew5c00913_si_001.pdf]

Supplementary information

New insights into the *in-situ* Fenton-like process by Copper(II)-hydroxylamine coupling: reactive species, applications and limitations

Simone Pellegrino[1], Pablo Martínez-Marco[2], Javier Moreno-Andrés[3], Esther Bautista-Chamizo[3], Ana María Amat[2], Claudio Minero[1], Enzo Laurenti[1], Iván Sciscenko[1,†,], Marco Minella[1]*

1. Department of Chemistry, University of Turin, Via Pietro Giuria 7, 10125 Turin, Italy.
2. Textile and Paper Engineering Department, Universitat Politècnica de València, Plaza Ferrándiz y Carbonell S/N, 03801 Alcoy, Spain.
3. Department of Environmental Technologies, Faculty of Marine and Environmental Sciences, INMAR-Marine Research Institute, University of Cádiz, Campus Universitario Puerto Real, 11510 Puerto Real, Cádiz, Spain.

*Corresponding author, Iván Sciscenko: ivanmatias.sciscenko@unito.it

† Previous institution: Textile and Paper Engineering Department, Universitat Politècnica de València, Plaza Ferrándiz y Carbonell S/N, 03801 Alcoy, Spain.

Number of pages: 20

Number of text sections: 3

Number of Tables: 4

Number of Figures: 17

Text S1. Orbitrap analysis conditions

High resolution mass spectrometry analysis were performed employing an Orbitrap IQ-X mass spectrometer (Thermo Fisher Scientific) equipped with a heated electrospray ionization source by direct infusion. The ion source was operated in positive and negative ion mode (for DMPO and BA transformation product analysis, respectively) using the following settings: spray voltage of 3.3 kV (+), ion transfer tube temperature of 275 °C and vaporizer temperature of 290 °C. N_2 was used as both sheath gas and auxiliary gas, with flow rates set at 40 and 10 arbitrary units (a.u.), respectively. The mass error (δ, difference between measured accurate mass and calculated accurate mass × 10^6) detected is < 1 ppm. All data were processed by FreeStyle software (v1.8 SP2, Thermo Fisher Scientific). For structural confirmation of compound, tandem mass spectrometry experiments (MS^2) were performed both by traditional collision induced dissociation (CID) and higher energy collisional dissociation (HCD) at a target resolution of 30,000 (at 200 m/z FWHM). CID and HCD techniques offer diverse fragmentation paths, ensuring accurate structural characterization when used together. Various normalized collision energies (NCE) ranging from 25 to 30 were examined.

Text S2. Moles of formed $NO_{(g)}$ and $NO_{2(g)}$ calculation

The calculation of formed gases was made integrating the area under the curves of $NO_{(x)}$ vs. time plots and assuming ideal gases situation. The room temperature was 25 ºC, whereas the employed pressure and flow rate from the synthetic air were 1 atm and 1.2 L min^{-1}, respectively. The $NO_{(x)}$ concentrations (measured every 5.5 s and expressed in ppm by the detector) were converted into mol knowing the aforementioned flow. Finally, a summatory within the desired period of time ($t_i = i + 5.5$ s, with $i = 0$ – 3602.5 s) was calculated to obtain the formed mol of $NO_{(x)}$. The following equation resumes the required calculation:

$$nNO_{(x)} = \sum_i \frac{C_i}{10^6}\frac{VP}{RT} = \sum_i \frac{C_i}{10^6} 0.0045\, mol \qquad \text{Eq. 2}$$

Where C_i are the $NO_{(x)}$ concentration and 0.0045 mol the number of total mol of gas in the aforementioned 5.5 s measurement time intervals.

Text S3. Benzoate oxidation by $^1O_2$: bimolecular rate constant determination

The BA bimolecular rate constant oxidation by $^1O_2$ ($k_{^1O_2+BA}$) was measured according to Eq. 1 (deduced from steady state approximation) as performed in a previous work.[1] The $^1O_2$ was generated by irradiating with yellow light a solution containing the probe (BA 10 µM or FFA 80 µM) and Rose Bengal 10 µM (the photosensitiser). The employed lamp was a Philips TL-D 18W 1SL/25, 700 lumens, $\lambda_{max} = 590$ nm.

$$k_{^1O_2+\text{probe}} = -\frac{k'_{d,^1O_2}}{t\cdot R_{f,^1O_2}}\cdot \ln\frac{[probe]_t}{[probe]_0} \qquad \text{Eq. 1}$$

Firstly, the $^1O_2$ formation rate ($R_{f,^1O_2}$) was calculated by measuring the degradation rate of FFA 80 µM, which was ca. 70% in 1 h ($\ln([FFA]_t/[FFA]_0)/t = 3 \times 10^{-4}$ s^{-1}). Since the bimolecular rate constant between FFA and $^1O_2$ is known ($k_{^1O_2+FFA} = 1.2 \times 10^8$ M^{-1} s^{-1}),[2] as well as the deactivation rate constant of $^1O_2$ in water ($k'_{d,^1O_2} = 2.5 \times 10^5$ s^{-1}),[2] the singlet oxygen formation rate can be obtained, being $R_{f,^1O_2} = 6.25\times10^{-7}$ M s^{-1}. When repeating the procedure with the solution containing BA 10 µM and Rose Bengal 10 µM, no appreciable BA degradation was observed (< 5% in 24 h), thus, the bimolecular rate constant between $^1O_2$ and BA should be < 2 × 10^5 M^{-1} s^{-1} (value obtained assuming 5% BA degradation in 24 h, i.e., $\ln([BA]_t/[BA]_0)/t = 5.9 \times 10^{-7}$ s^{-1}). Analogous results were obtained when increasing the BA initial concentration to 20 µM or reducing it to 4 µM (the Rose Bengal concentration was always fixed to 10 µM).

Table S1. Simulated wastewater composition. The resulting pH was of 7.8±0.2. Values based on related works.[3–5]

Compound	Concentration (µM)
$CaSO_4$	1500
$MgSO_4$	500
KCl	500
$NaNO_3$	3000
NaH_2PO_4	400
$NaHCO_3$	5000
$CuSO_4$	10
$FeCl_3$	50
$MnSO_4$	100
Na_2MoO_4	0.5
$ZnSO_4$	5
$Na_2B_4O_7$	2.5
EDTA	5
Humic acids	35 (mg L^{-1})

Table S2. Thermodynamical equilibrium constants of Cu(II) (20 - 25 ºC). *Only reported by van den Berg (1984)[6] at pH 9 and ionic strength of 0.7 mol kg^{-1} (far from the working conditions of this work) with a value of logK = 3.5.

Reaction	Reported constant	Ionic strength (mol kg^{-1})	Reference
$NH_3OH^+ + H_2O \rightleftharpoons NH_2OH + H_3O^+$	$-logK_a = 6.02$	0.0	Elsewhere
$CO_2{\cdot}H_2O + H_2O \rightleftharpoons HCO_3^- + H_3O^+$	$-logK_{a1} = 6.4$	0.0	
$HCO_3^- + H_2O \rightleftharpoons CO_3^{2-} + H_3O^+$	$-logK_{a1} = 10.3$	0.0	
$H_2PO_4^- + H_2O \rightleftharpoons HPO_4^{2-} + H_3O^+$	$-logK_{a2} = 7.2$	0.0	
$HPO_4^{2-} + H_2O \rightleftharpoons PO_4^{3-} + H_3O^+$	$-logK_{a2} = 12.4$	0.0	
$H_3BO_3 + H_2O \rightleftharpoons B(OH)_4^- + H_3O^+$	$-logK_{a1} = 9.2$	0.0	
$HEDTA + H_2O \rightleftharpoons EDTA + H_3O^+$	$-logK_{a5} = 6.16$	0.0	
$Cu^{2+} + NH_2OH \rightleftharpoons Cu(NH_2OH)^{2+}$	$logK_f < 2.4$	0.1	Szilárd (1963)[7]
$Cu^{2+} + NH_3OH^+ \rightleftharpoons Cu(NH_3OH)^{3+}$	$logK_f = 2.5$		
$Cu^{2+} + H_2O \rightleftharpoons Cu(OH)^+ + H^+$	$-logK_a = 7.95$	0.0	Powell et al. (2007)[8]
$Cu^{2+} + 2\ H_2O \rightleftharpoons Cu(OH)_{2(aq)} + H^+$	$-log\beta = 16.5$		
$Cu^{2+} + 2\ OH^- \rightleftharpoons Cu(OH)_{2(s)}$	$-logK_{sp} = 19.3$		
$Cu^{2+} + 2\ OH^- \rightleftharpoons CuO_{(s)} + H_2O$	$-logK_{sp} = 20.4$		
$Cu^{2+} + HCO_3^- \rightleftharpoons CuHCO_3^+$	$logK_f = 1.84$		

$Cu^{2+} + CO_3^{2-} \rightleftharpoons CuCO_{3(aq)}$	$\log K_f = 6.75$		
$Cu^{2+} + CO_3^{2-} \rightleftharpoons CuCO_{3(s)}$	$\log K_{sp} = -11.5$		
$Cu^{2+} + 2\ CO_3^{2-} \rightleftharpoons Cu(CO_3)_2^{2-}$	$\log K_f = 10.3$		
$2\ Cu^{2+} + CO_3^{2-} + 2\ OH^- \rightleftharpoons Cu_2(CO_3)(OH)_{2(s)}$	$\log K_{sp} = -33.16$		
$Cu^{2+} + HPO_4^{2-} \rightleftharpoons CuHPO_{4(aq)}$	$\log K_f = 3.3$		
$3\ Cu^{2+} + 2\ PO_4^{3-} \rightleftharpoons Cu_3(PO_4)_{2(s)}$	$\log K_{sp} = -36.9$		
$Cu^{2+} + SO_4^{2-} \rightleftharpoons CuSO_{4\ (aq)}$	$\log K_f = 2.3$		
$Cu^{2+} + Cl^- \rightleftharpoons CuCl^+$	$\log K_f = 0.8$		
$Cu^{2+} + H_3BO_3 \rightleftharpoons CuH_2BO_3^+ + H^+$	*	---	---
$Cu^{2+} + NO_3^- \rightleftharpoons CuNO_3^+$	$\log K_f = 0.5$	0.0	Hutchinson et al. (1973)[9]
$Cu^{2+} + EDTA \rightleftharpoons Cu^{II}$-EDTA	$\log K_f = 18.7$	0.1	Bucheli-Witschel and Egli (2001)[10]

Table S3. Major compounds detected by high resolution mass spectrometry during the oxidation of DMPO 1.8 mM by Cu(II)/NH_2OH, Cu(II)/H_2O_2, Cu(II)/NH_2OH/H_2O_2, NH_2OH/H_2O_2 (positive mode).

m/z (g mol^{-1}), z = +1	Molecular formula	δ (ppm)	Tentative molecular structure
114.0912 ([DMPOH]$^+$)	$C_6H_{12}ON$	-1.35	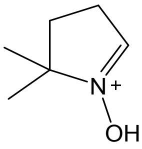
121.9661 (impurity)	C_2H_5ONCu	-0.52	---
128.0706 ([DMPOHX]$^+$)	$C_6H_{10}O_2N$	-0.02	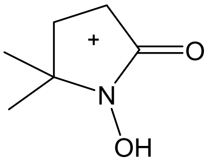
130.0863 ([DMPOH-OH]$^+$)	$C_6H_{12}O_2N$	0.02	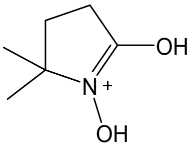
136.0733 ([DMPONa]$^+$)	$C_6H_{11}ONNa$	0.02	---
144.1019 ([DMPOH-OCH_3]$^+$ or [DMPOH-CH_2OH]$^+$)	$C_7H_{14}O_2N$	0.06	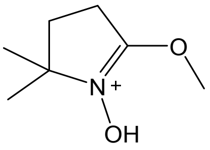

			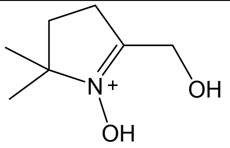
144.9821 (impurity)	$C_4H_6N_2Cu$	-0.24	$Cu(CH_3CN)_2^+$
227.1754 (DMPO dimer)	$C_{12}H_{23}O_2N_2$	0.11	---

Table S4. Major compounds detected by high resolution mass spectrometry during the oxidation of benzoic acid 50 μM by Cu(II)/NH_2OH (negative mode).

m/z (g mol^{-1}), z = -1	Molecular formula	δ (ppm)	Tentative molecular structure
121.0294 (benzoate)	$C_7H_5O_2$	-1.13	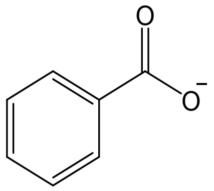
122.0246	$C_6H_4O_2N$	-1.33	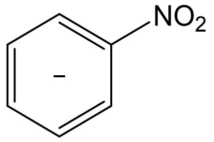
125.0244	$C_6H_5O_3$	-0.76	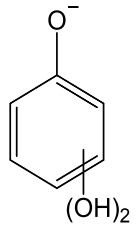
137.0240	$C_7H_5O_3$	-1.5	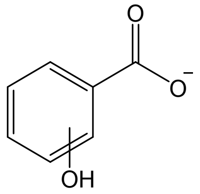
153.0192	$C_7H_5O_4$	-1.59	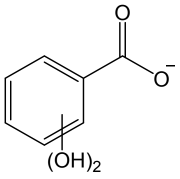

166.0146	$C_7H_4O_4N$	-0.24	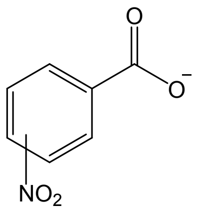

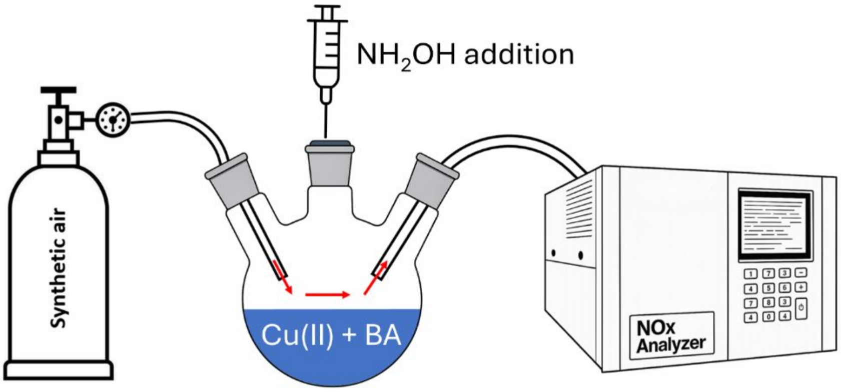

Figure S1. Scheme of the employed set-up for the $NO_{(g)}$ and $NO_{2(g)}$ detection in gaseous phase by Cu(II)/NH_2OH with different initial benzoate concentrations.

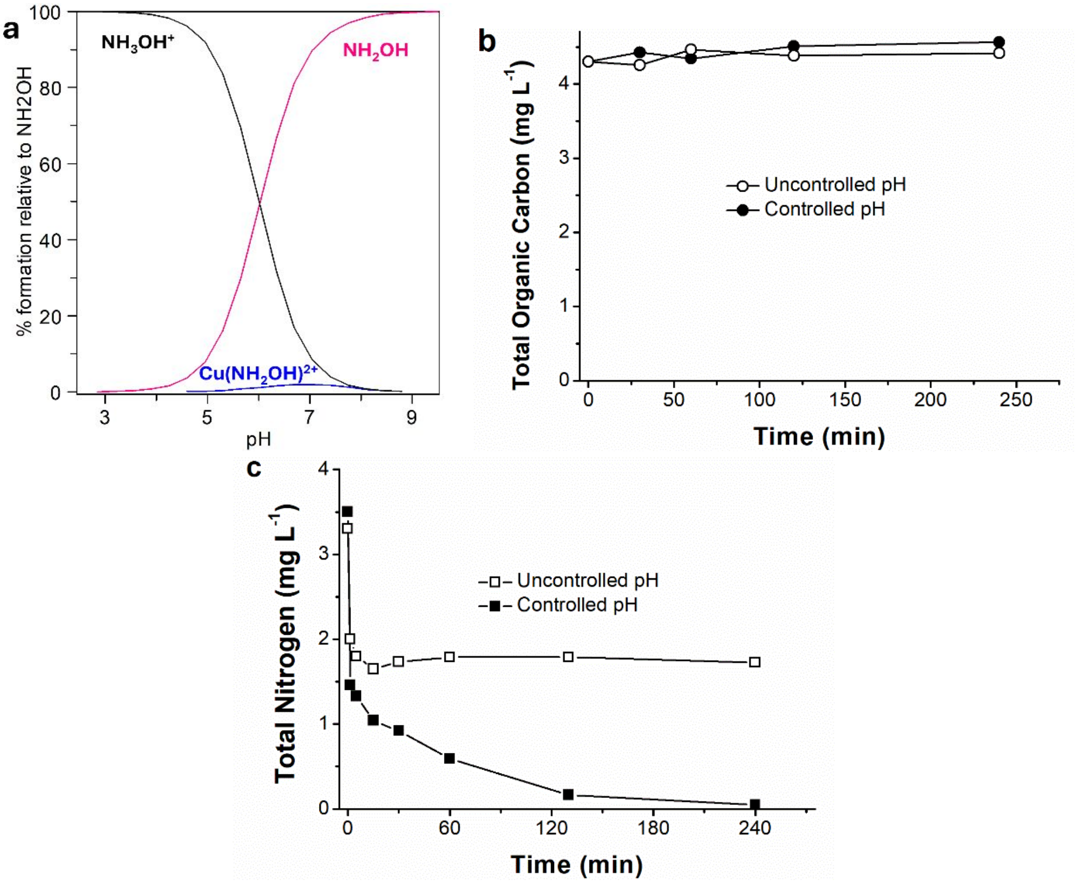

Figure S2. a) speciation diagram of NH_2OH 250 µM in the presence of Cu(II) 100 µM; b) and c) total organic carbon and nitrogen decay over time, respectively, during the Cu(II)/NH_2OH process at uncontrolled and controlled pH 7.0 (i.e. without and with the NaOH 0.1 M continuous dosage after NH_2OH addition, respectively). Conditions, $[NH_2OH]_0$ = 250 µM and $[Cu(II)]_0$ = 100 µM.

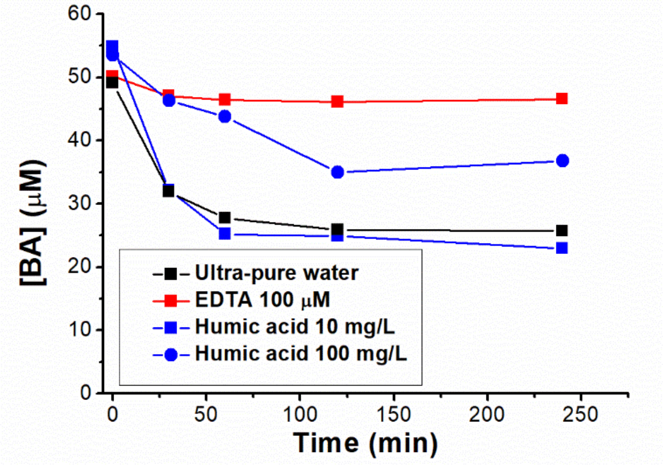

Figure S3. Effect of competing ligands for Cu(II): effect of EDTA 100 µM and humic acid, 10 and 100 mg L^{-1}, respectively, during the degradation of benzoate by Cu(II)/NH_2OH process. Conditions: $[Cu(II)]_0$ = 100 µM, $[NH_2OH]_0$ = 250 µM, pH_0 = 7.0.

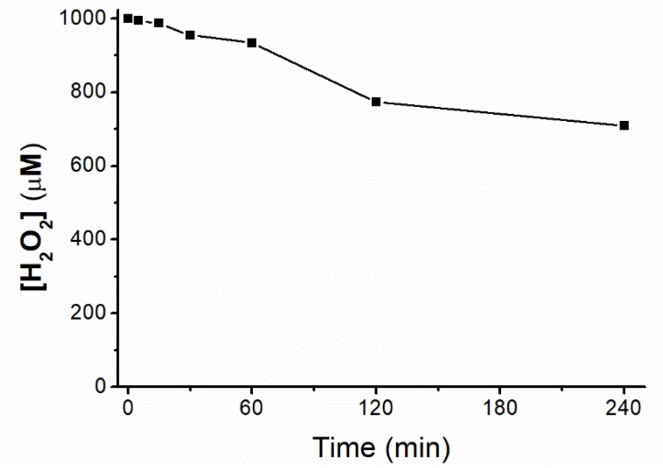

Figure S4. H_2O_2 decomposition kinetics by Cu(II)/NH_2OH/H_2O_2. Conditions: $[H_2O_2]_0$ = 1 mM, $[Cu(II)]_0$ = 100 µM, $[NH_2OH]_0$ = 250 µM, pH_0 = 7.0.

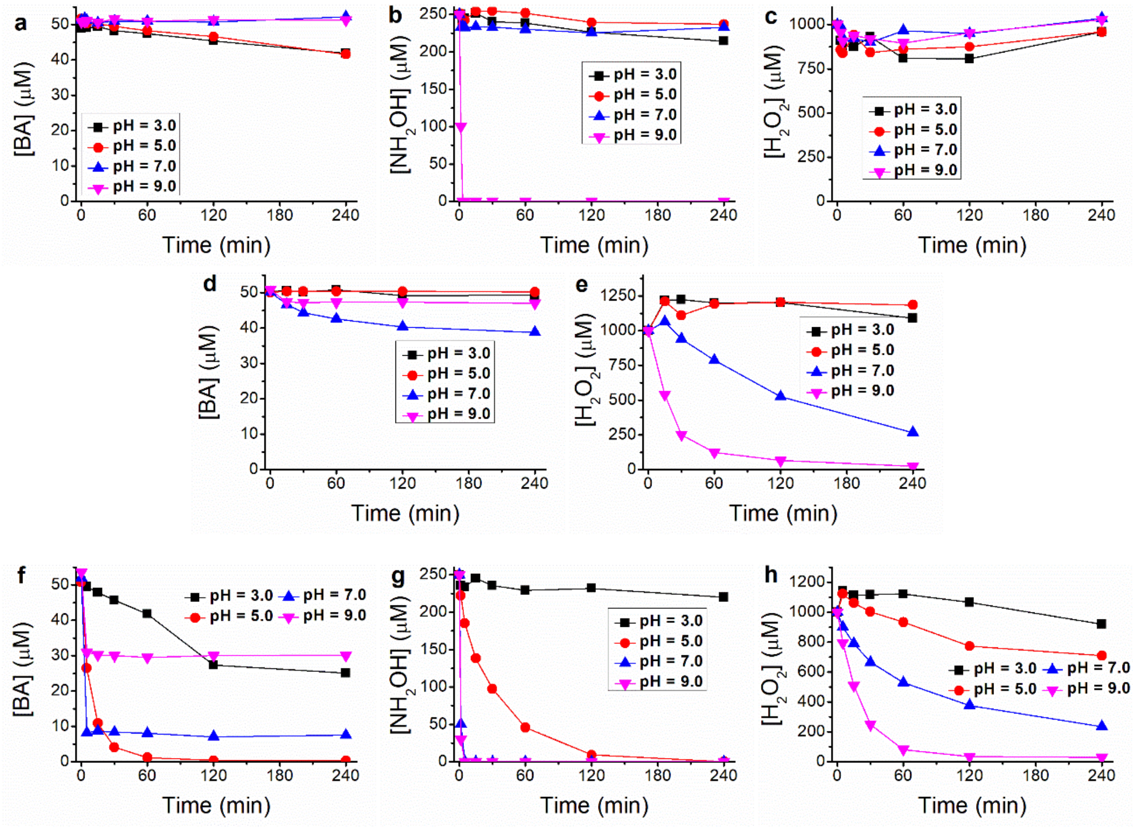

Figure S5. BA, NH_2OH and H_2O_2 kinetics for the different studied processes at fixed pH values: a-c) NH_2OH/H_2O_2 process, d-e) Cu(II)/H_2O_2 process, and f-h) Cu(II)/NH_2OH/H_2O_2 process. Conditions: $[H_2O_2]_0 = 1$ mM, $[Cu(II)]_0 = 100$ μM, $[NH_2OH]_0 = 250$ μM.

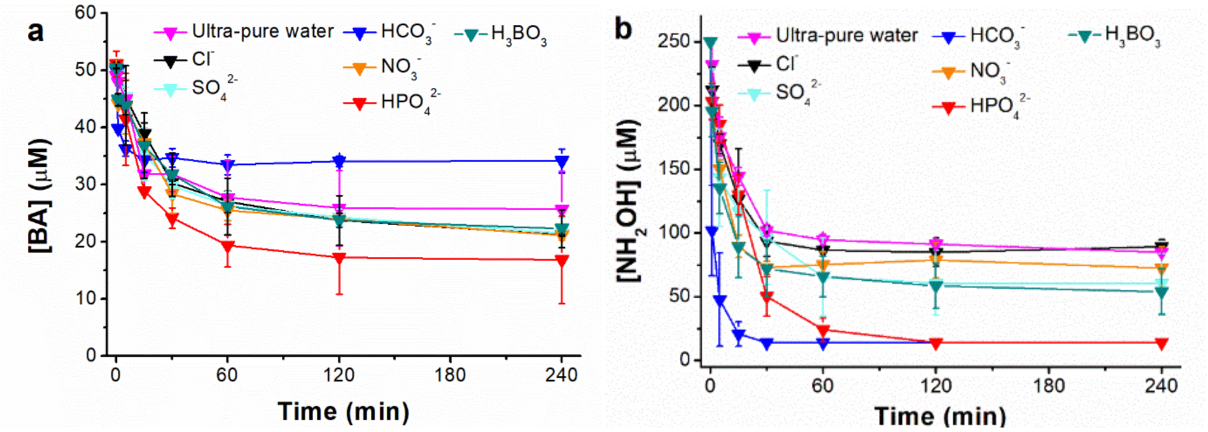

Figure S6. Effect of anions on the Cu(II)/NH_2OH performance ($pH_0 = 7.0$; $[Cu(II)]_0 = 100$ μM; $[NH_2OH]_0 = 250$ μM): a) BA and b) NH_2OH concentration decay over time with different anions (1 mM each), respectively.

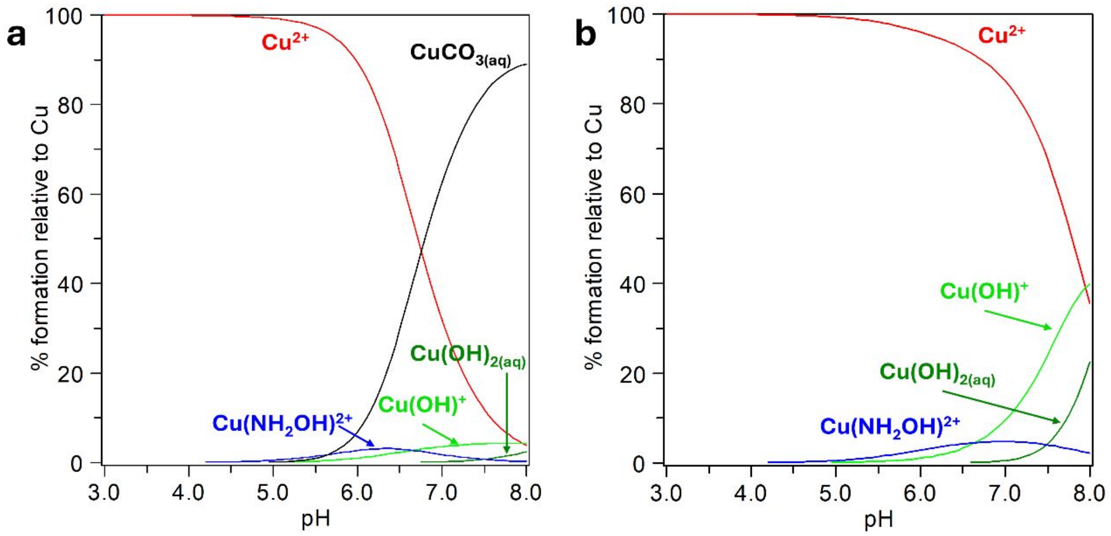

Figure S7. Cu(II) 100 µM speciation in the presence of: a) NH_2OH 250 µM and HCO_3^- 1 mM, b) NH_2OH 250 µM and HPO_4^{2-} 1 mM.

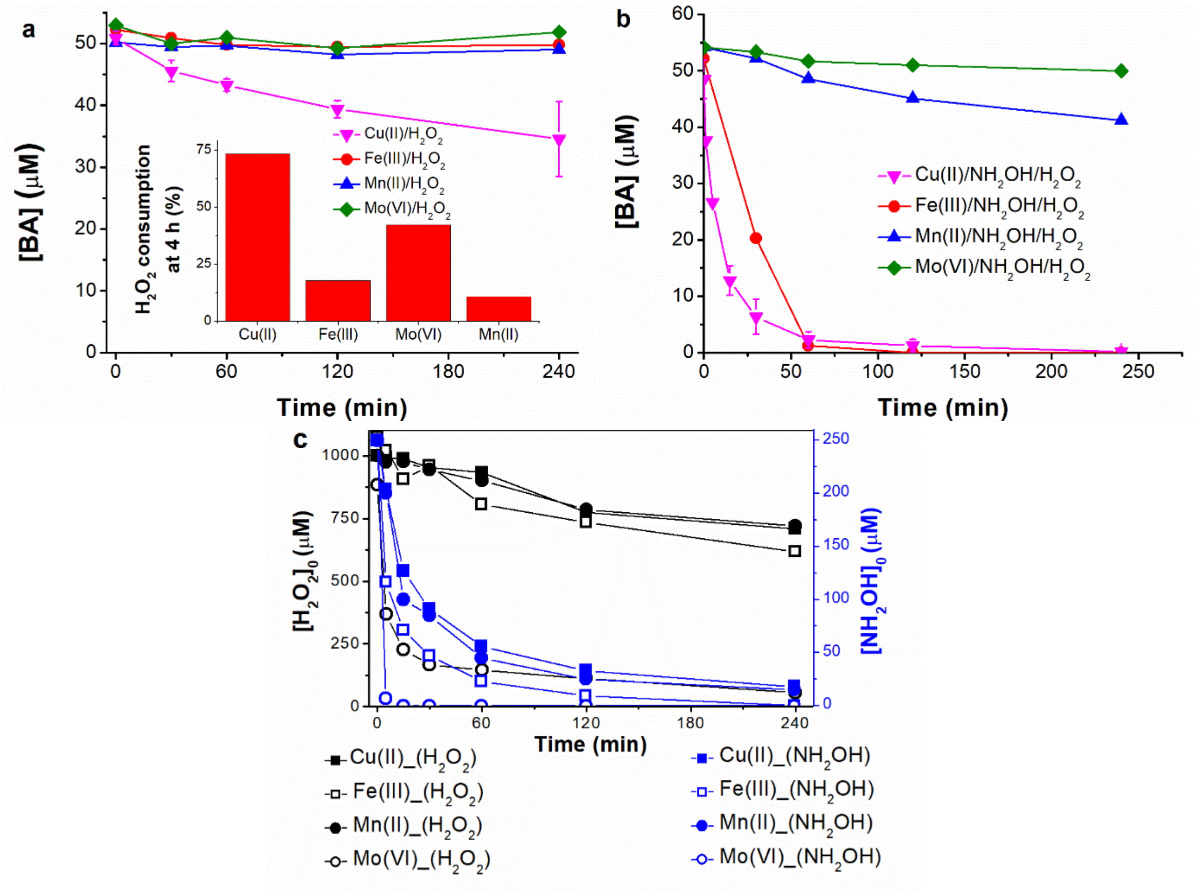

Figure S8. Results from the addition of Fe(III), Mn(II) and Mo(VI) into H_2O_2 and NH_2OH/H_2O_2 (without Cu(II)): a) BA oxidation by Fe(III)/H_2O_2, Mn(II)/H_2O_2, and Mo(VI)/H_2O_2, respectively, with the corresponding H_2O_2 consumption after 4 h of process (inset, consumption by Cu(II)/H_2O_2 placed as reference); b) BA and c) H_2O_2 and NH_2OH kinetics obtained by Fe(III)/H_2O_2/NH_2OH, Mn(II)/H_2O_2/NH_2OH, and Mo(VI)/H_2O_2/NH_2OH treatments, respectively (in c) the results from

Cu(II)/NH_2OH/H_2O_2 were also added to facilitate the comparison). Conditions: $[H_2O_2]_0$ = 1 mM, $[Fe(III)]_0$ = 100 μM, $[Mn(II)]_0$ = 100 μM, $[Mo(VI)]_0$ = 100 μM, $[NH_2OH]_0$ = 250 μM, pH_0 = 7.0.

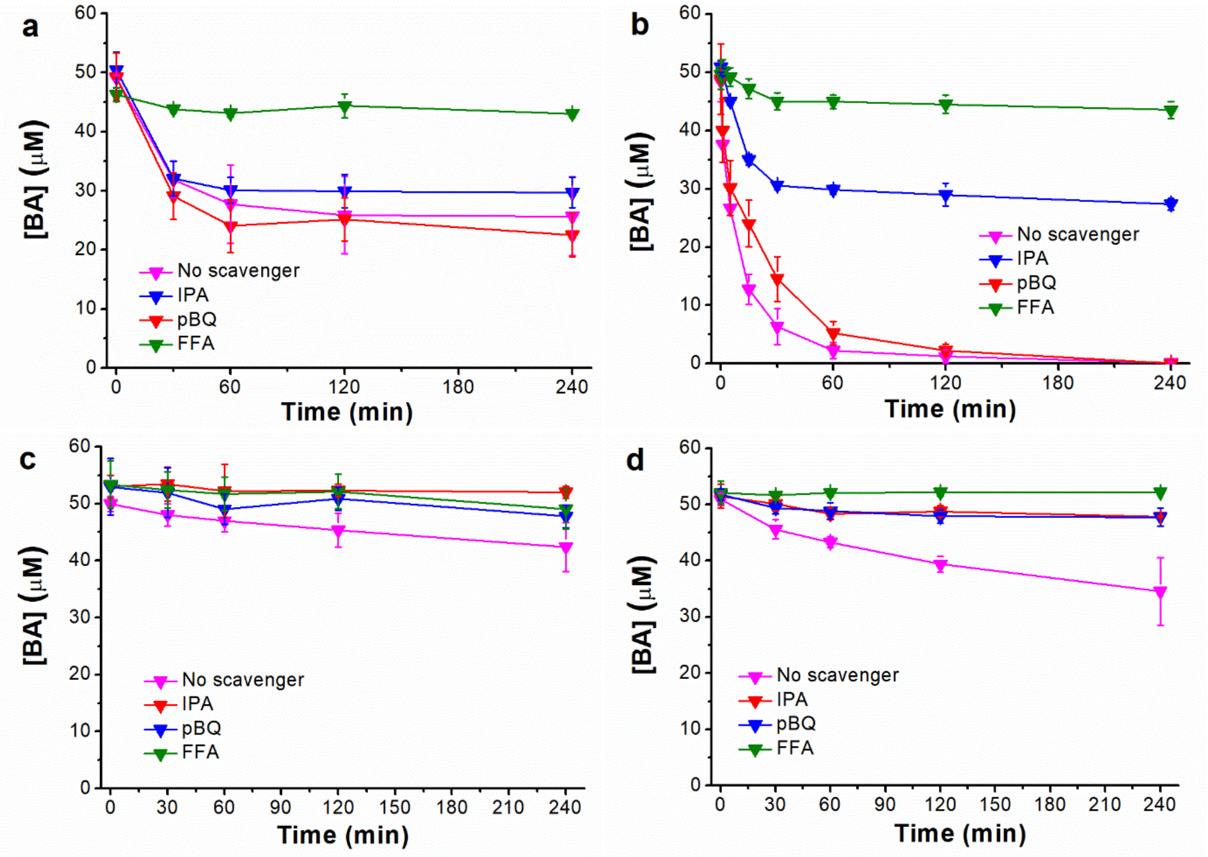

Figure S9. Effect of scavengers, p-benzoquinone (pBQ, red), isopropyl alcohol (IPA, blue), and furfuryl alcohol (FFA, green) on the BA degradation performance by: a) Cu(II)/NH_2OH, b) Cu(II)/NH_2OH/H_2O_2, c) H_2O_2/NH_2OH, and d) Cu(II)/H_2O_2.

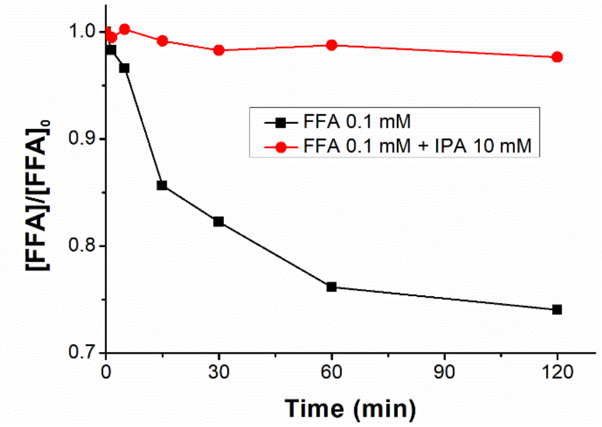

Figure S10. Furfuryl alcohol degradation kinetics by Cu(II)/NH_2OH in the presence and absence of IPA excess. Conditions: pH_0 = 7.0, $[Cu(II)]_0$ = 100 μM, $[NH_2OH]_0$ = 250 μM, $[FFA]_0$ = 100 μM, $[IPA]_0$ = 10 mM.

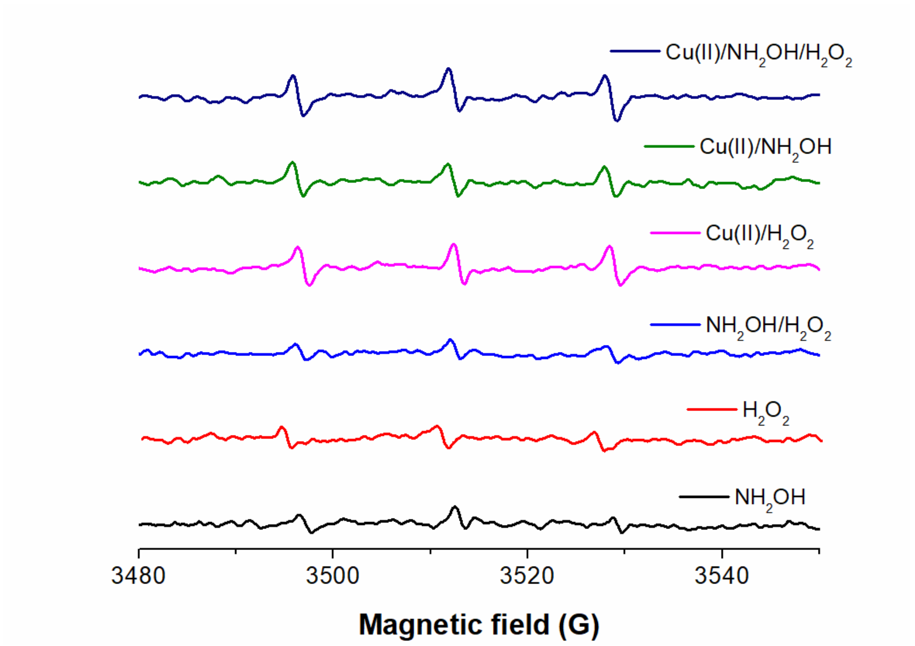

Figure S11. EPR spectra obtained by the stated situations after 5 min of mixing in the presence of TEMP 77 mM. Conditions: $pH_0 = 7.0$, $[Cu(II)]_0 = 100$ µM, $[NH_2OH]_0 = 250$ µM, $[H_2O_2]_0 = 1$ mM.

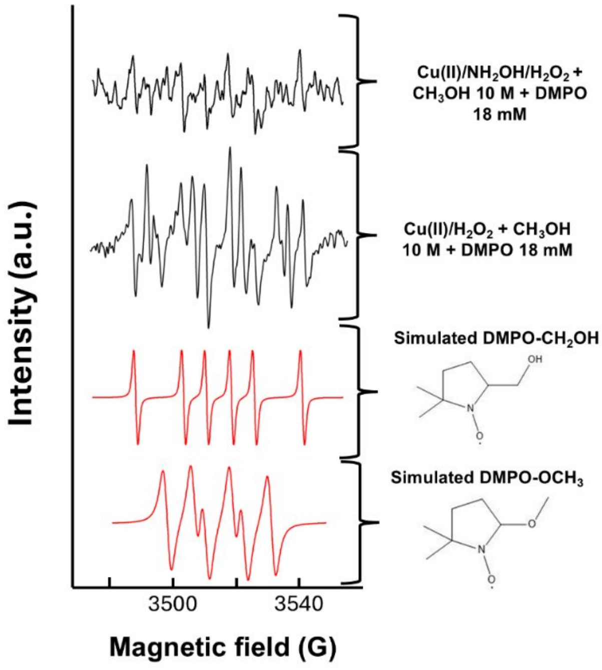

Figure S12. Cu(III) detection tests at the EPR: Cu(II)/H_2O_2, with and without NH_2OH, reactions in the presence of DMPO 18 mM and methanol 10 M. Conditions: $pH_0 = 7.0$, $[Cu(II)]_0 = 100$ µM, $[NH_2OH]_0 = 250$ µM, $[H_2O_2]_0 = 1$ mM.

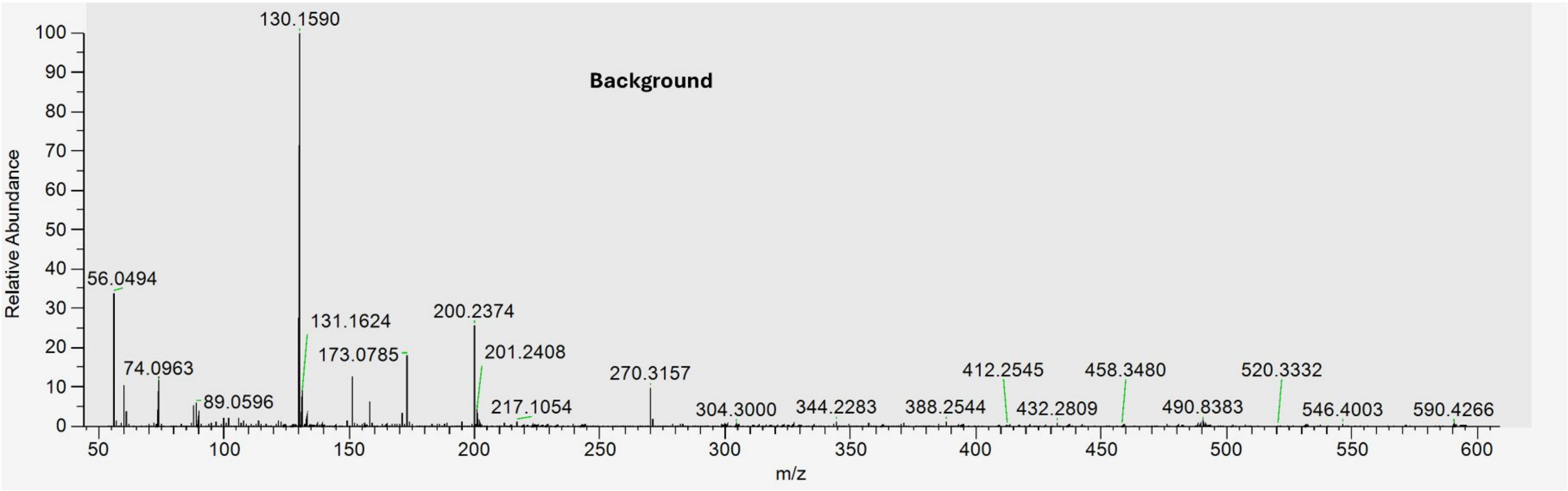
Background
Relative Abundance
m/z
130.1590
56.0494
131.1624
200.2374
173.0785
201.2408
74.0963
270.3157
89.0596
217.1054
304.3000
344.2283
388.2544
412.2545
432.2809
458.3480
490.8383
520.3332
546.4003
590.4266

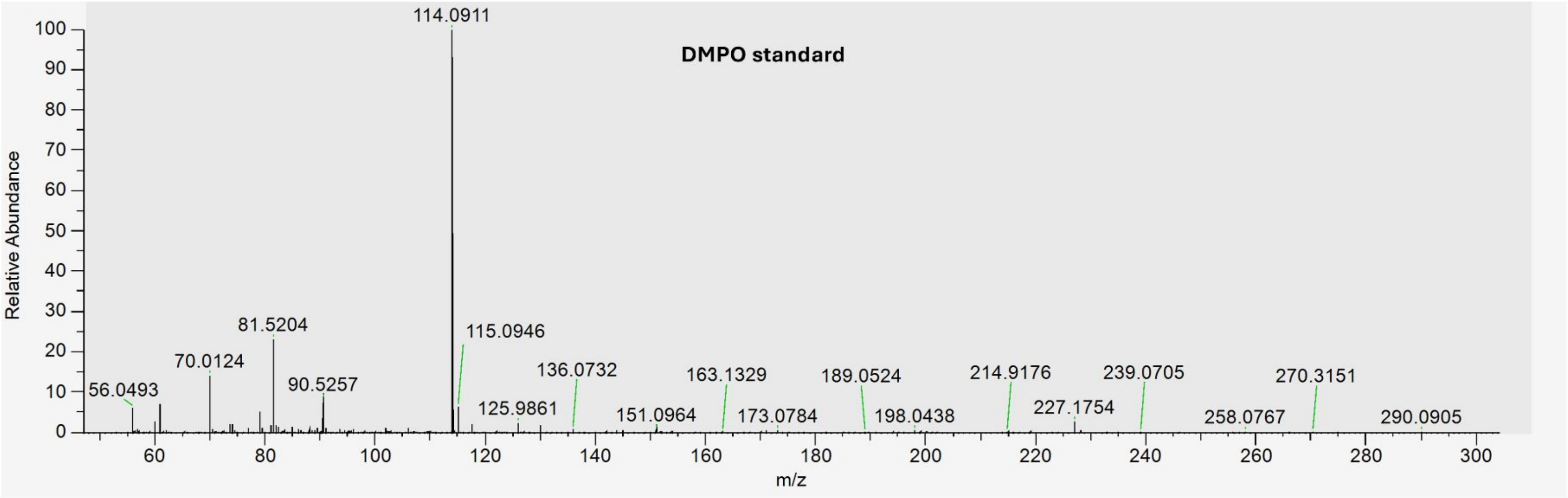
DMPO standard
Relative Abundance
m/z
114.0911
81.5204
115.0946
70.0124
90.5257
56.0493
136.0732
125.9861
163.1329
151.0964
173.0784
189.0524
198.0438
214.9176
227.1754
239.0705
258.0767
270.3151
290.0905

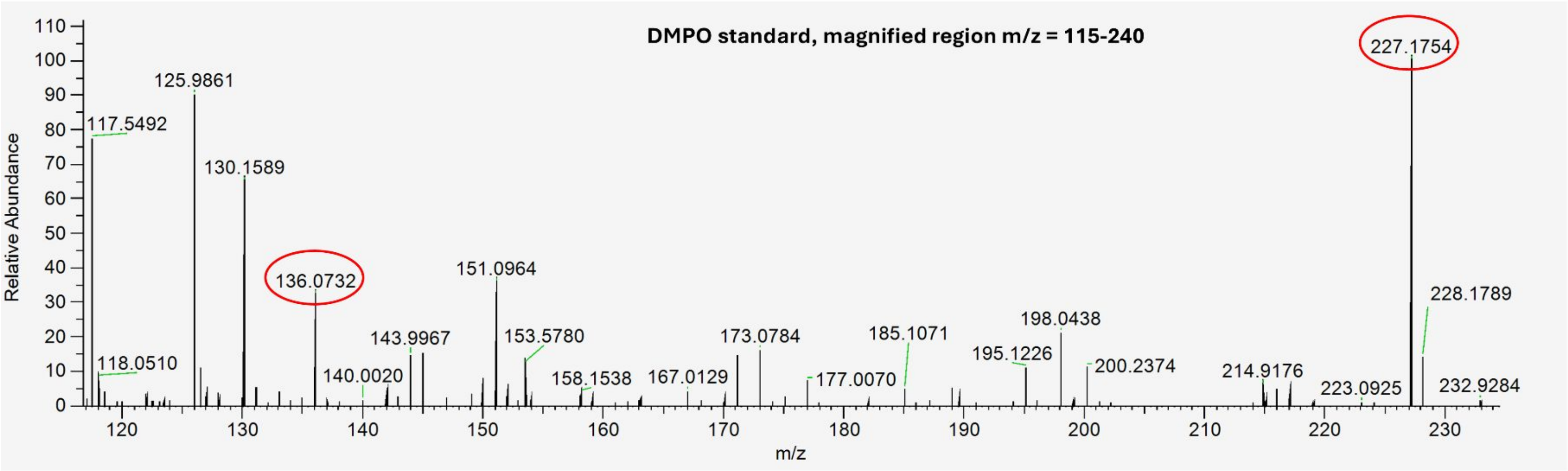
DMPO standard, magnified region m/z = 115-240
227.1754
125.9861
117.5492
130.1589
136.0732
151.0964
143.9967
153.5780
173.0784
185.1071
198.0438
228.1789
195.1226
118.0510
140.0020
158.1538
167.0129
177.0070
200.2374
214.9176
223.0925
232.9284
Relative Abundance
m/z

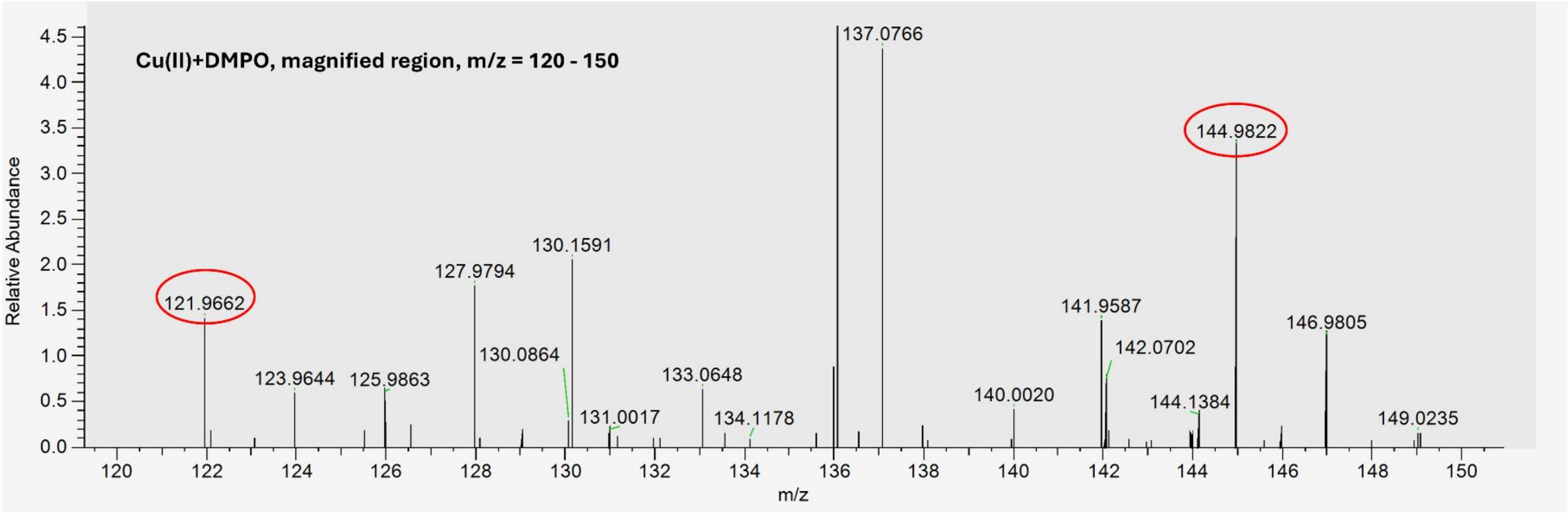
Cu(II)+DMPO, magnified region, m/z = 120 - 150
137.0766
144.9822
130.1591
127.9794
121.9662
141.9587
146.9805
142.0702
130.0864
133.0648
123.9644
125.9863
140.0020
144.1384
131.0017
134.1178
149.0235
Relative Abundance
m/z

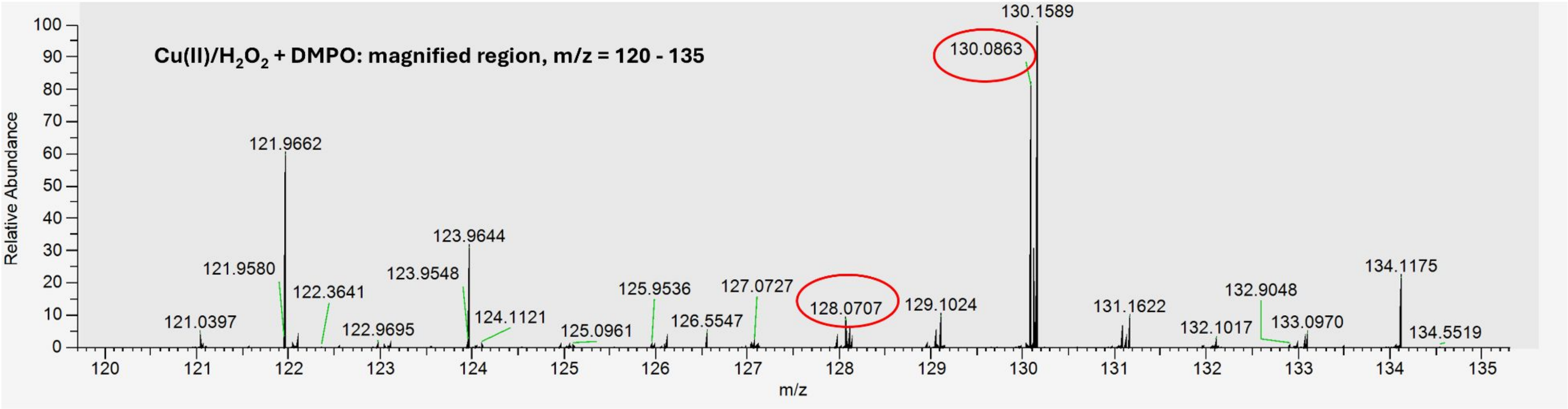

Cu(II)/H2O2 + DMPO: magnified region, m/z = 120 - 135
Relative Abundance
m/z
130.1589
130.0863
121.9662
123.9644
121.9580
123.9548
122.3641
125.9536
127.0727
128.0707
129.1024
131.1622
132.9048
134.1175
121.0397
124.1121
126.5547
122.9695
125.0961
132.1017
133.0970
134.5519

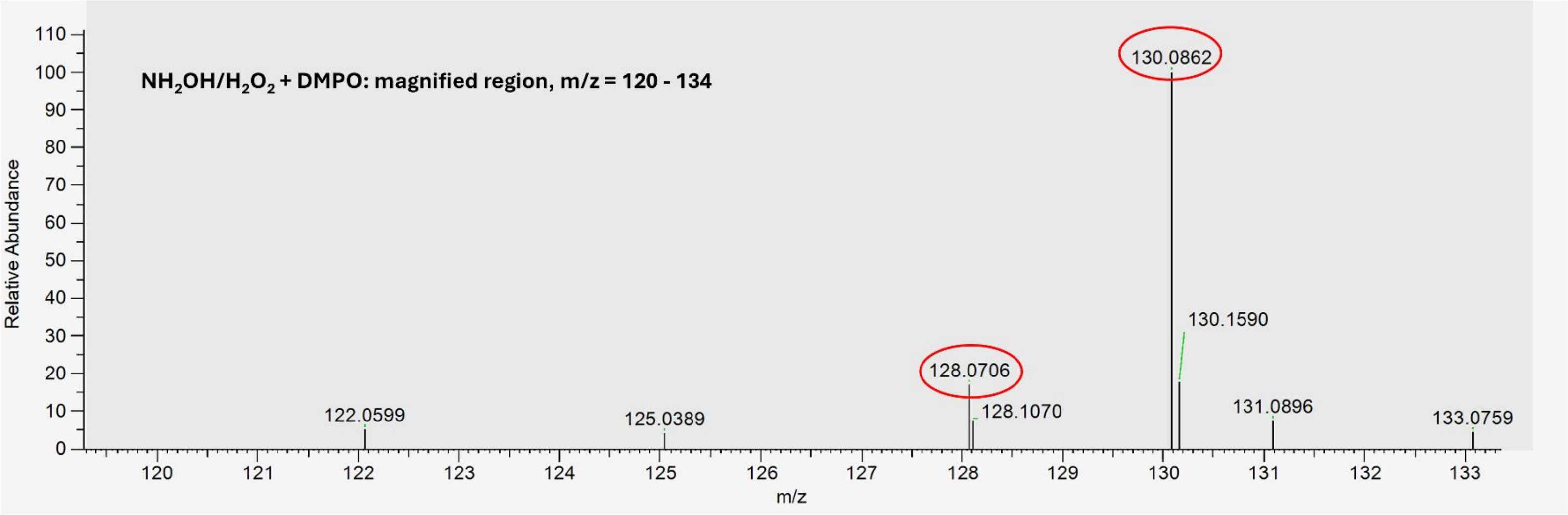

NH2OH/H2O2 + DMPO: magnified region, m/z = 120 - 134
Relative Abundance
m/z
130.0862
130.1590
128.0706
128.1070
131.0896
122.0599
125.0389
133.0759

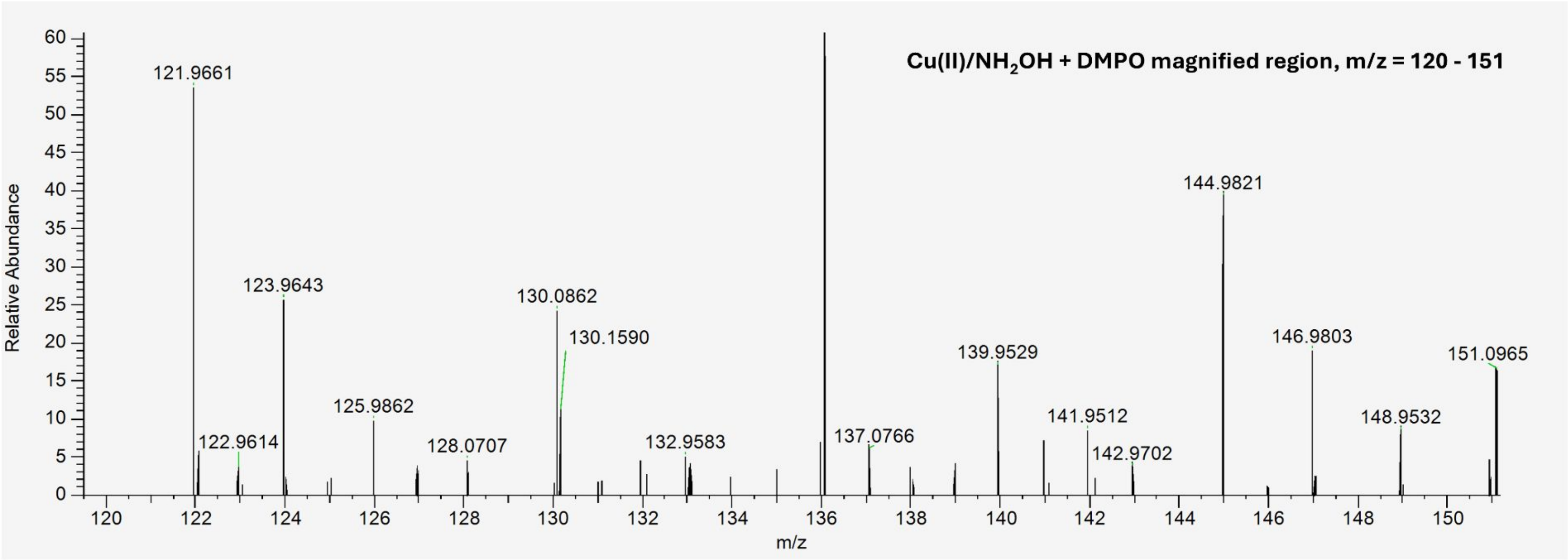

Cu(II)/NH2OH + DMPO magnified region, m/z = 120 - 151
Relative Abundance
m/z
121.9661
122.9614
123.9643
125.9862
128.0707
130.0862
130.1590
132.9583
137.0766
139.9529
141.9512
142.9702
144.9821
146.9803
148.9532
151.0965

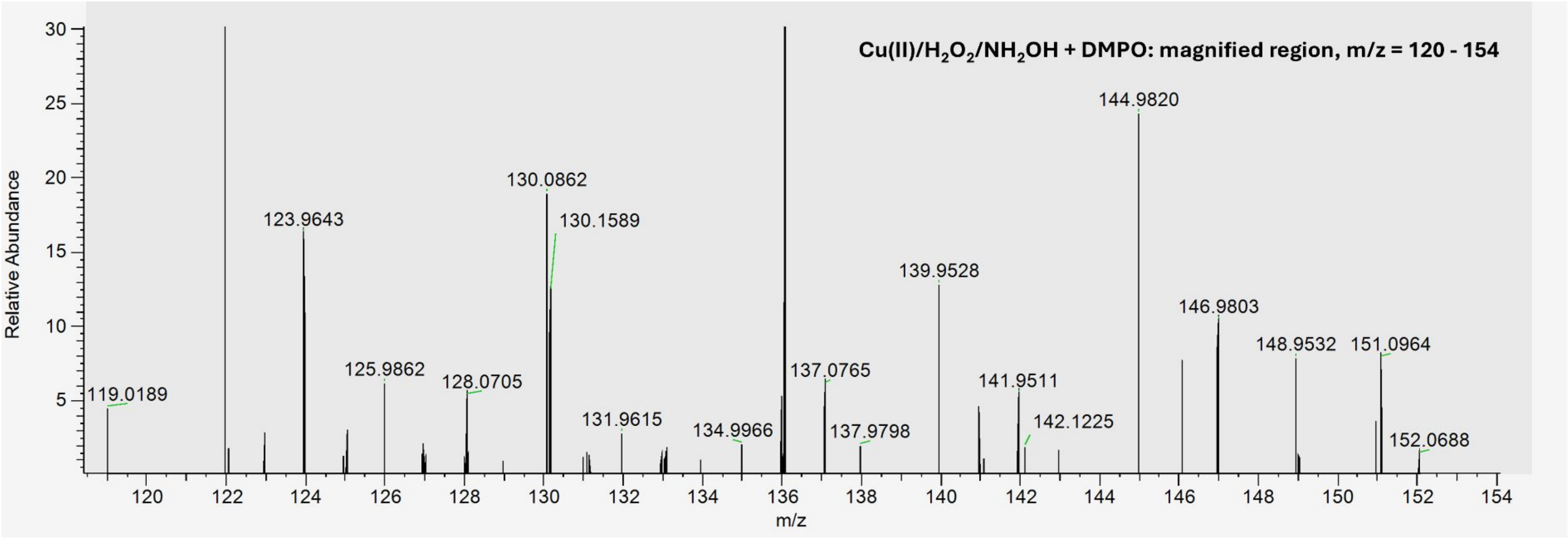

Cu(II)/H2O2/NH2OH + DMPO: magnified region, m/z = 120 - 154
Relative Abundance
m/z
119.0189
123.9643
125.9862
128.0705
130.0862
130.1589
131.9615
134.9966
137.0765
137.9798
139.9528
141.9511
142.1225
144.9820
146.9803
148.9532
151.0964
152.0688

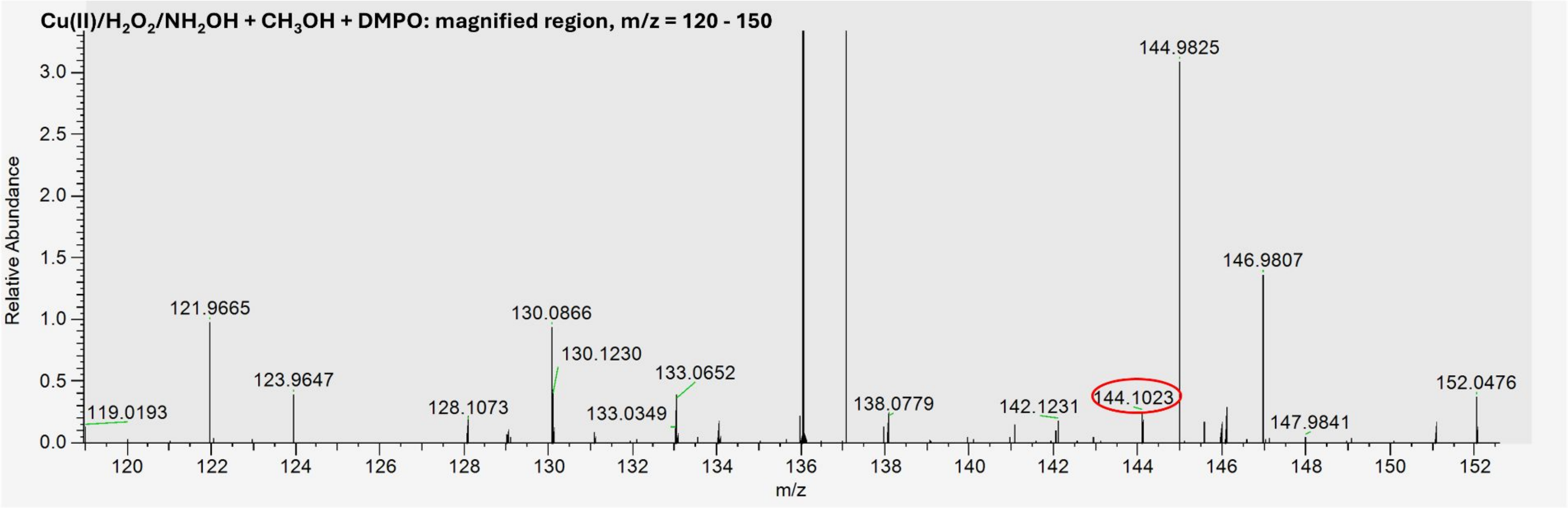

Figure S13 (pages S13-S17). Obtained spectrograms for the specified cases: oxidation of DMPO 1.8 mM (positive mode). Conditions: pH_0 = 7.0, $[Cu(II)]_0$ = 100 µM, $[NH_2OH]_0$ = 250 µM, $[H_2O_2]_0$ = 1 mM.

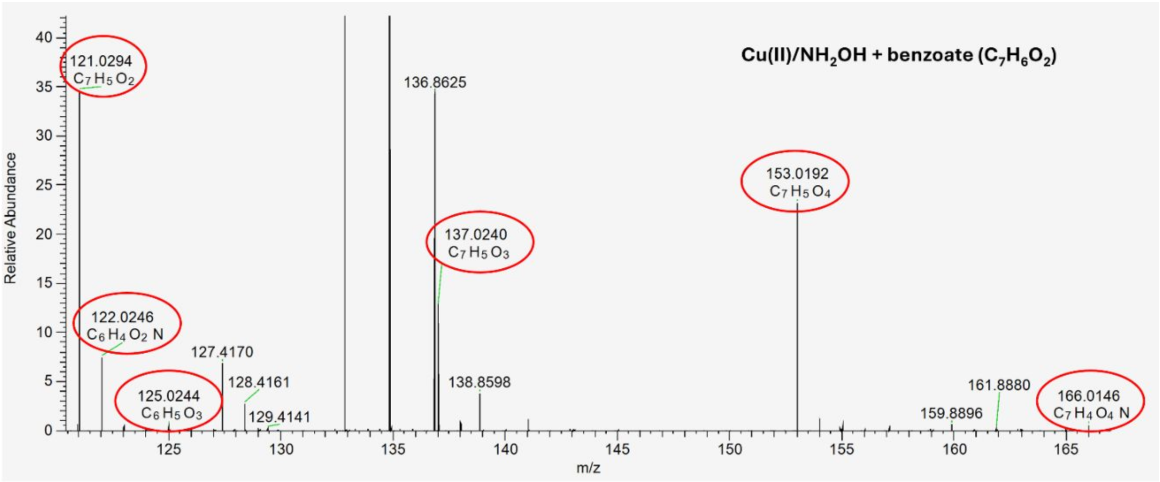

Figure S14. Transformation products of benzoate by Cu(II)/NH_2OH after 2 h of treatment (negative mode). Conditions: $pH_0 = 7.0$, $[Cu(II)]_0 = 100$ μM, $[NH_2OH]_0 = 250$ μM, $[BA]_0 = 50$ μM.

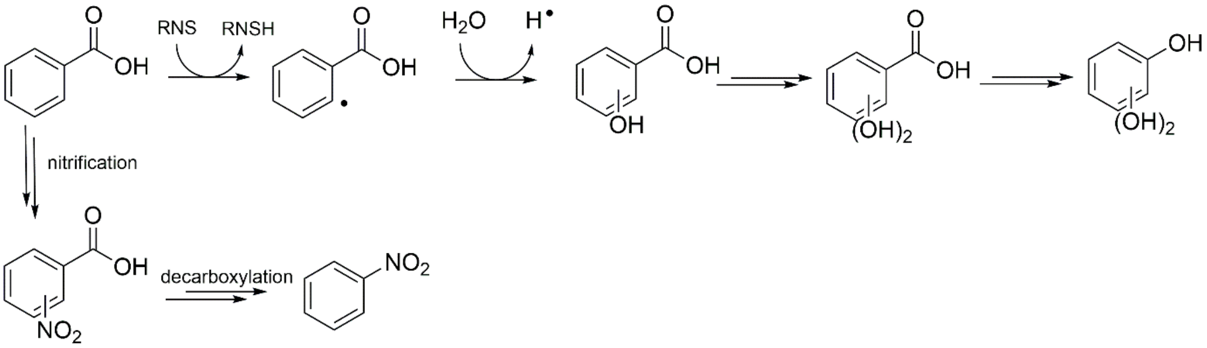

Figure S15. Proposed degradation pathway of benzoate.

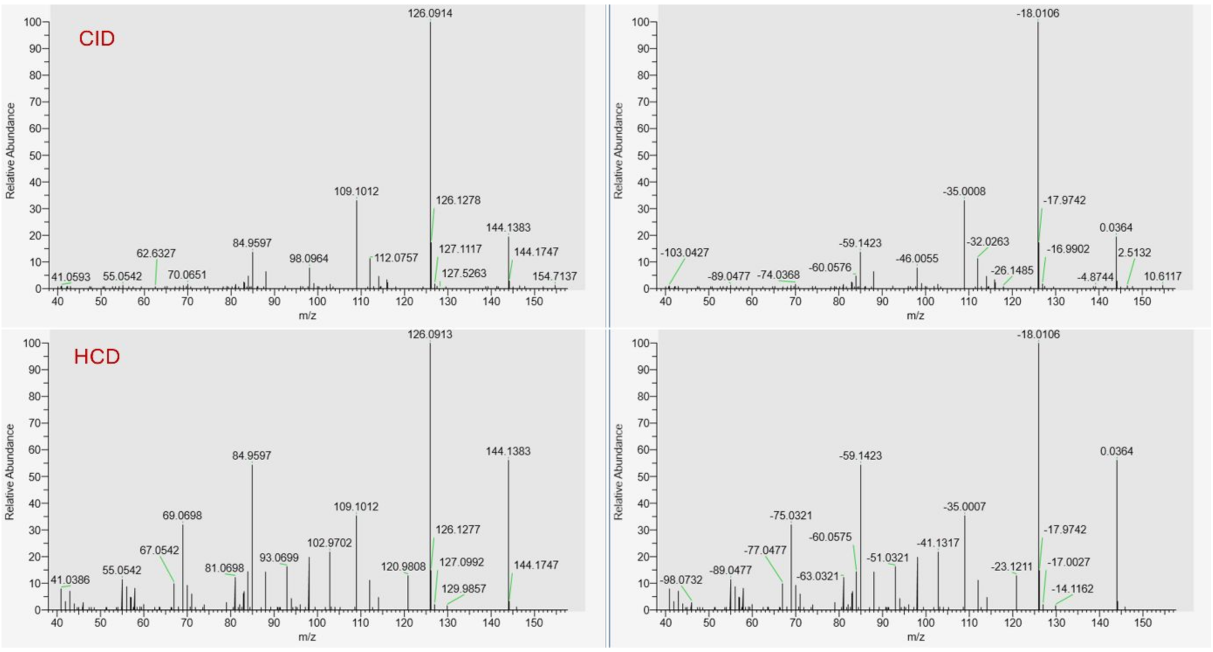

Figure S16. Fragmentation signals from $C_7H_{14}O_2N$ (DMPO-OCH_3 or DMPO-CH_2OH, m/z = 144.1023) by CID and HCD. Conditions: $pH_0 = 7.0$, $[Cu(II)]_0 = 100$ μM, $[NH_2OH]_0 = 250$ μM, $[CH_3OH]_0 = 10$ M, $[DMPO]_0 = 1.8$ mM.

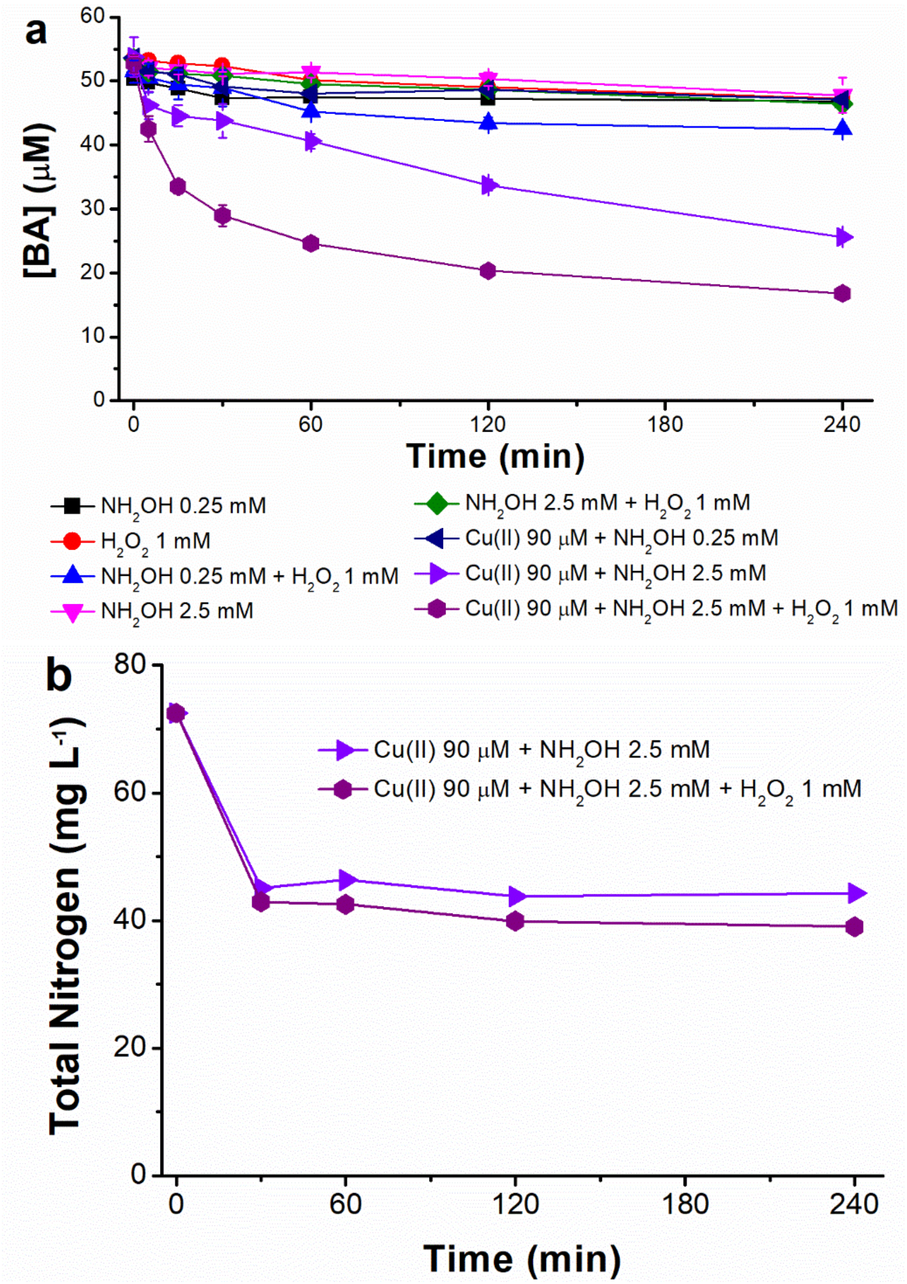

Figure S17. Results obtained in simulated wastewater (containing 10 μM of Cu(II)): a) benzoate oxidation kinetics with the addition of the stated reagents, and b) total nitrogen kinetics when employing Cu(II) 90 μM + NH_2OH 2.5 mM with and without H_2O_2 1 mM.

References

(1) Carena, L.; Minella, M.; Barsotti, F.; Brigante, M.; Milan, M.; Ferrero, A.; Berto, S.; Minero, C.; Vione, D. Phototransformation of the Herbicide Propanil in Paddy Field Water. *Environ. Sci. Technol.* **2017**, *51* (5), 2695–2704. https://doi.org/10.1021/acs.est.6b05053.

(2) Haag, W. R.; Hoigné, J.; Gassman, E.; Braun, A. M. Singlet Oxygen in Surface Waters - Part I: Furfuryl Alcohol as a Trapping Agent. *Chemosphere* **1984**, *13* (5–6), 631–640. https://doi.org/10.1016/0045-6535(84)90199-1.

(3) Sciscenko, I.; Mora, M.; Micó, P.; Escudero-Oñate, C.; Oller, I.; Arques, A. EEM-PARAFAC as a Convenient Methodology to Study Fluorescent Emerging Pollutants Degradation: (Fluoro)Quinolones Oxidation in Different Water Matrices. *Sci. Total Environ.* **2022**, *852* (April), 158338. https://doi.org/10.1016/j.scitotenv.2022.158338.

(4) Kumar, R. R.; Cho, J. Y. Reuse of Hydroponic Waste Solution. *Environ. Sci. Pollut. Res.* **2014**, *21* (16), 9569–9577. https://doi.org/10.1007/s11356-014-3024-3.

(5) Dey, M. G.; Langenfeld, N. J.; Bugbee, B. Copper Can Be Elevated in Hydroponics and Peat-Based Media for Potential Disease Suppression: Concentration Thresholds for Lettuce and Tomato. *HortScience* **2023**, *58* (4), 459–468. https://doi.org/10.21273/HORTSCI17048-22.

(6) van den Berg, C. M. G. Speciation of Boron with Cu2+, Zn2+, Cd2+ and Pb2+ in 0.7 M KNO3 and in Sea-Water. *Geochim. Cosmochim. Acta* **1984**, *48* (12), 2613–2617. https://doi.org/10.1016/0016-7037(84)90309-0.

(7) Szilárd, I. Stability Constants of Metal Ion - Hydroxylamine Complexes in Aqueous Solution. *Acta Chem. Scand.* **1963**, *17*, 2674–2680. https://doi.org/10.3891/acta.chem.scand.17-2674.

(8) Powell, K. J.; Brown, P. L.; Byrne, R. H.; Gajda, T.; Hefter, G.; Sjöberg, S.; Wanner, H. Chemical Speciation of Environmentally Significant Metals with Inorganic Ligands Part 2: The Cu2+-OH-, Cl-, CO32-, SO42-, and PO43- Systems (IUPAC Technical Report). *Pure Appl. Chem.* **2007**, *79* (5), 895–950. https://doi.org/10.1351/pac200779050895.

(9) Hutchinson, M. H.; Higginson, W. C. E. Stability Constants for Association between Bivalent Cations and Some Univalent Anions. *J. Chem. Soc. Dalt. Trans.* **1973**, No. 12, 1247. https://doi.org/10.1039/dt9730001247.

(10) Bucheli-Witschel, M.; Egli, T. Environmental Fate and Microbial Degradation of Aminopolycarboxylic Acids. *FEMS Microbiol. Rev.* **2001**, *25* (1), 69–106. https://doi.org/10.1016/S0168-6445(00)00055-3.
